# Nucleic Acid Specificity, Cellular Localization and Reduced Toxicities of Thiazole Orange‐Neomycin Conjugates

**DOI:** 10.1002/open.202400189

**Published:** 2024-12-27

**Authors:** Antwine W. McFarland, Lawrence P. Fernando, Patrick Kellish, Sandra P. Story, Gretchen B. Schober, Sunil Kumar, Changjun Gong, Ada King, Xianchang Gong, Alain S. Leutou, Dev P. Arya

**Affiliations:** ^1^ NUBAD LLC Greer 29650 USA; ^2^ Department of Chemistry Clemson University Clemson, SC 29634 USA

**Keywords:** Thiazole Orange, Neomycin, RNA binder, Fluorescent probe, Cell staining

## Abstract

Selective binding of small molecule ligands to nucleic acids with high affinity and limited toxicity remains an important goal in the development of compounds that can probe DNA or RNA in cells. Thiazole orange is a cell semi‐permeant, fluorescent cyanine dye, with low background noise, that binds several forms of nucleic acids. However, thiazole orange can exhibit cytotoxicity when used at high concentration and/or with prolonged exposure. Neomycin is a non‐fluorescent antibiotic with affinity for several forms of nucleic acids, but does not readily enter mammalian cells. Conjugation of neomycin with thiazole orange can exploit the properties of each individual compound, yielding a small molecule that could be used for nontoxic application in cellular analysis by microscopic imaging. We demonstrate that conjugation of neomycin with thiazole orange increases the cell permeability of neomycin, decreases the cytotoxicity of thiazole orange, and exhibits a greater degree of intracellular RNA targeted localization in the nucleolus, when compared to thiazole orange. Relative to thiazole orange, the conjugated compounds showed a much higher degree of stabilization of the nucleic acids as reflected in a greater denaturation temperature. Ultimately, our studies indicate that the conjugated thiazole orange‐neomycin compounds can be used as an RNA targeted, less cytotoxic alternative for cellular labeling.

## Graphical abstract

The conjugation of neomycin with thiazole orange is shown to increase the cell permeability of neomycin, decrease the cytotoxicity of thiazole orange, and exhibit a greater degree of intracellular RNA targeted localization in the nucleolus, when compared to thiazole orange. These thiazole orange‐neomycin conjugates can be used as an RNA targeted, less cytotoxic alternative for cellular labeling.

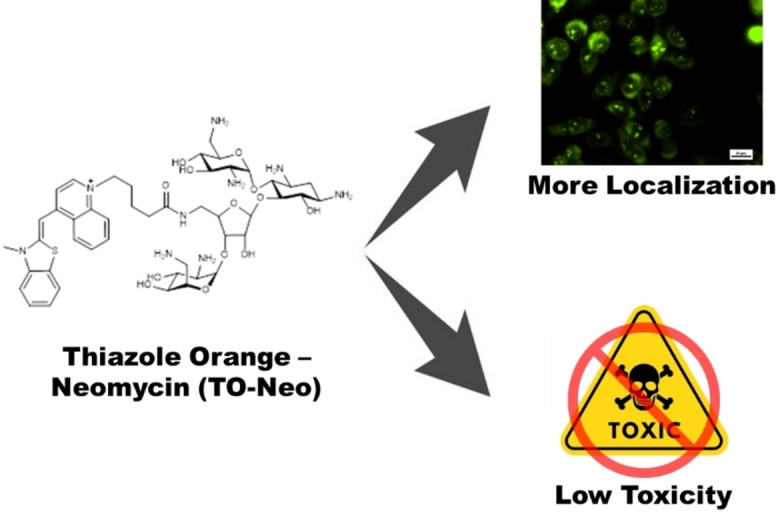

## Introduction

Development of small molecule ligands capable of binding RNA or DNA with selectivity, high efficacy, and limited toxicity is an active field of research. Small molecule probes targeting nucleic acid should ideally have the following three basic properties; 1) the ability to target specific nucleic acid sequences within specific cellular locations such as the nucleolus or mitochondria, 2) the ability to have a signal that is selectively turned on when bound to the target, and 3) have a relatively low background to signal ratio when free in solution.[^1^, ^2^]

Thiazole orange (TO) is a cell semi‐permeant, fluorescent cyanine dye that can bind with different forms of nucleic acids with selectivity for RNA over DNA.^[3]^ The popularity of TO as a fluorophore is due to its predominantly quenched relative fluorescence intensity when exposed to water as an unbound fluorophore and bright relative fluorescence intensity when water is displaced upon binding to its nucleic acid target. Binding serves to restrict intramolecular torsional movement and acts to “turn‐on” the relative fluorescence intensity signal of TO.[^2^, ^4^] Several molecular probes have been developed utilizing TO to detect a variety of biomolecules and metal ions.[^2^, ^4^] In addition to its binding to cellular DNA and RNA duplexes, TO can also bind with tetramolecular oligonucleotides capable of forming G quadruplex structures that resemble telomeric sequences to report on ligand interactions with G‐quadruplex DNA structures.[^5^, ^6^, ^7^] However, TO can cause cytotoxicity imparted by non‐specific intercalation with nuclear or mitochondrial DNA[^8^, ^9^, ^10^] indirectly causing transcription and regulatory interference^[11]^ or act as a photosensitizer when exposed to light with production of reactive oxygen species.^[12]^ Modification of TO via conjugation to other binding ligands can potentially lower the cytotoxic effect by limiting its non‐specific binding, while increasing its specificity towards particular sequences or structures.

Neomycin (NEO) is one such ligand for which the RNA and DNA binding properties are well understood. NEO belongs to the class of aminoglycoside antibiotics that acts by inhibiting prokaryotic protein synthesis by binding to the A‐site of the 16S ribosomal RNA in eubacteria.[^13^, ^14^] In prokaryotes, aminoglycosides such as NEO are taken up by energy‐dependent influx, however these molecules are poorly taken up in eukaryotic cells. NEO and other aminoglycosides can stabilize DNA and RNA triplexes, DNA⋅RNA hybrid duplexes/triplexes, poly‐A sequences, and DNA quadruplexes.[^15^, ^16^, ^17^, ^18^, ^19^] NEO is capable of binding A‐form (G−C rich) regions of DNA with a greater affinity over A−T rich DNA, as well as duplexed RNA.[^20^, ^21^]

The specific binding properties of aminoglycosides have been further explored by conjugating them with intercalators, with minor groove binders, and with oligonucleotides.[^19^, ^22^, ^23^, ^24^, ^25^, ^26^, ^27^, ^28^]

Considering the greater affinity of NEO for RNA and GC‐rich nucleic acid compared to TO,[^17^, ^18^, ^20^] we hypothesize that TO‐NEO conjugates would make for a more selective fluorescent probe, thus lowering toxicity and increasing specificity of TO, when linked to NEO.^[29]^ Our study demonstrates such benefits of conjugating NEO to TO. Two compounds, comprised of conjugated Thiazole Orange‐Neomycin (TO‐NEO) with two different linker lengths, were studied to explore the possibility of combining the potentially useful properties of these two molecules and to develop a fluorescent compound that could be used for nontoxic applications of cellular imaging either by flow cytometry and microscopic analysis.

**Figure 1:**
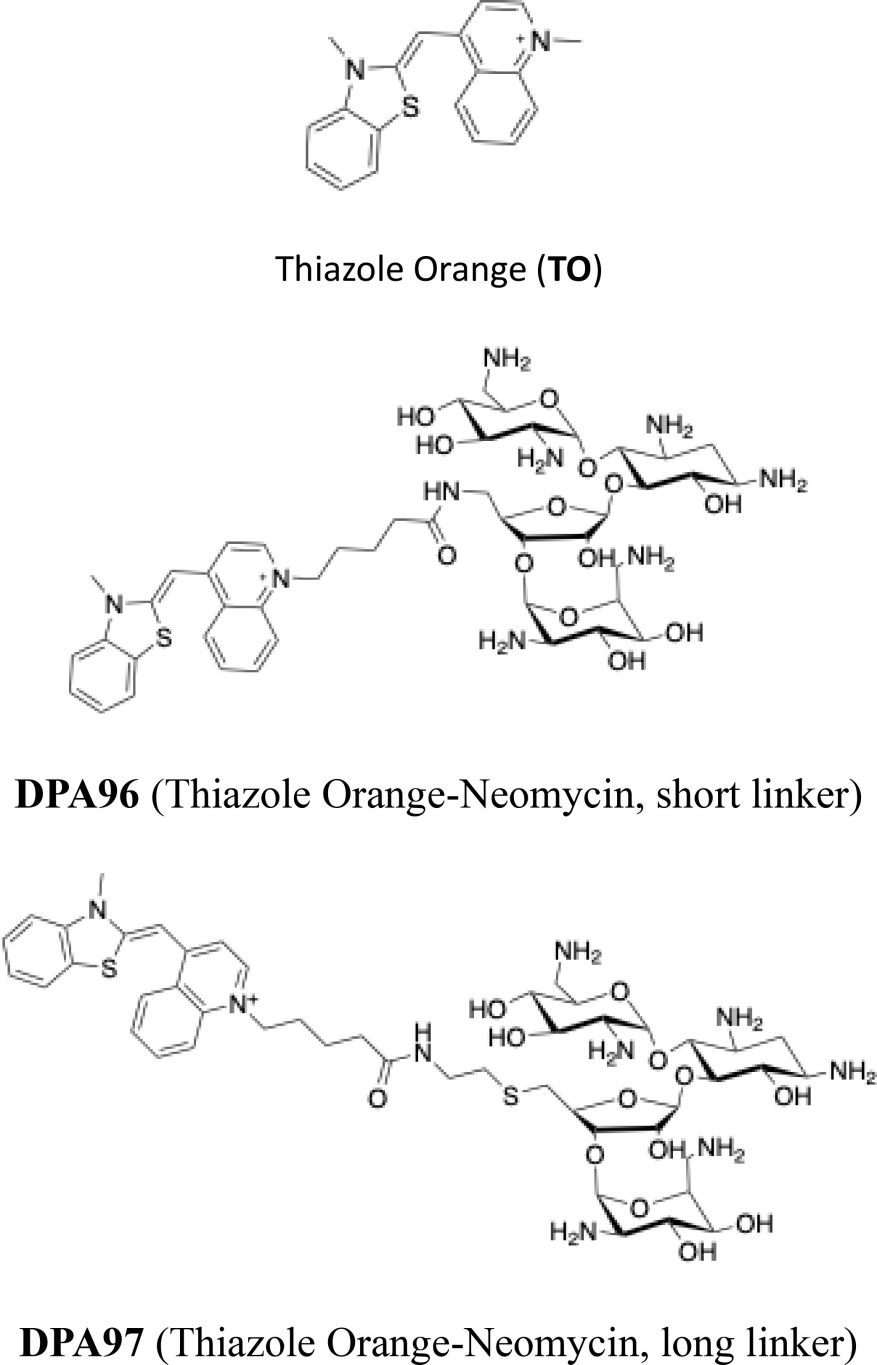
Chemical structures of TO and TO conjugated with NEO using a short (**DPA96**) and a longer linker (**DPA97**).

**Scheme 1:**
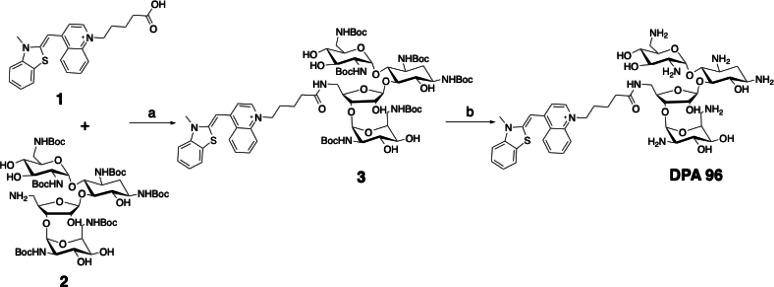
Synthetic scheme for preparation of DPA96. Reagents and conditions: (a) HATU, DIPEA, DMF, 24 h, rt. (b) TFA/dichloromethane (1:1), 30 min, rt.

The chemical structures of TO, DPA96 (TO‐NEO with short linker) and DPA97 (TO‐NEO with long linker) are displayed in Figure 1. The chemical scheme for synthesis of DPA96 can be seen in Scheme 1. Synthesis and characterization of **DPA97** has been previously reported.^[30]^ In the present study, our first objective was to investigate the influence of conjugation of TO to NEO, and probe linker length on fluorescence of various nucleic acids. This work included identifying GC rich DNA duplexes of interest that exhibited the largest observed change in relative fluorescence intensity upon TO‐NEO binding (ΔF), similar to published studies using unmodified TO.[^31^, ^32^] Secondly, the characterization of cellular uptake and intracellular localization was examined in several mammalian cell lines. Finally, cytotoxicity of the TO‐NEO conjugates as compared to TO was examined using cancerous MCF7; DU 145 cells and non‐cancerous HEK‐293 cells (Scheme 1).

## Results and Discussion

### Relative Fluorescence Intensity Characterization of TO‐NEO Conjugates

**Figure 2:**
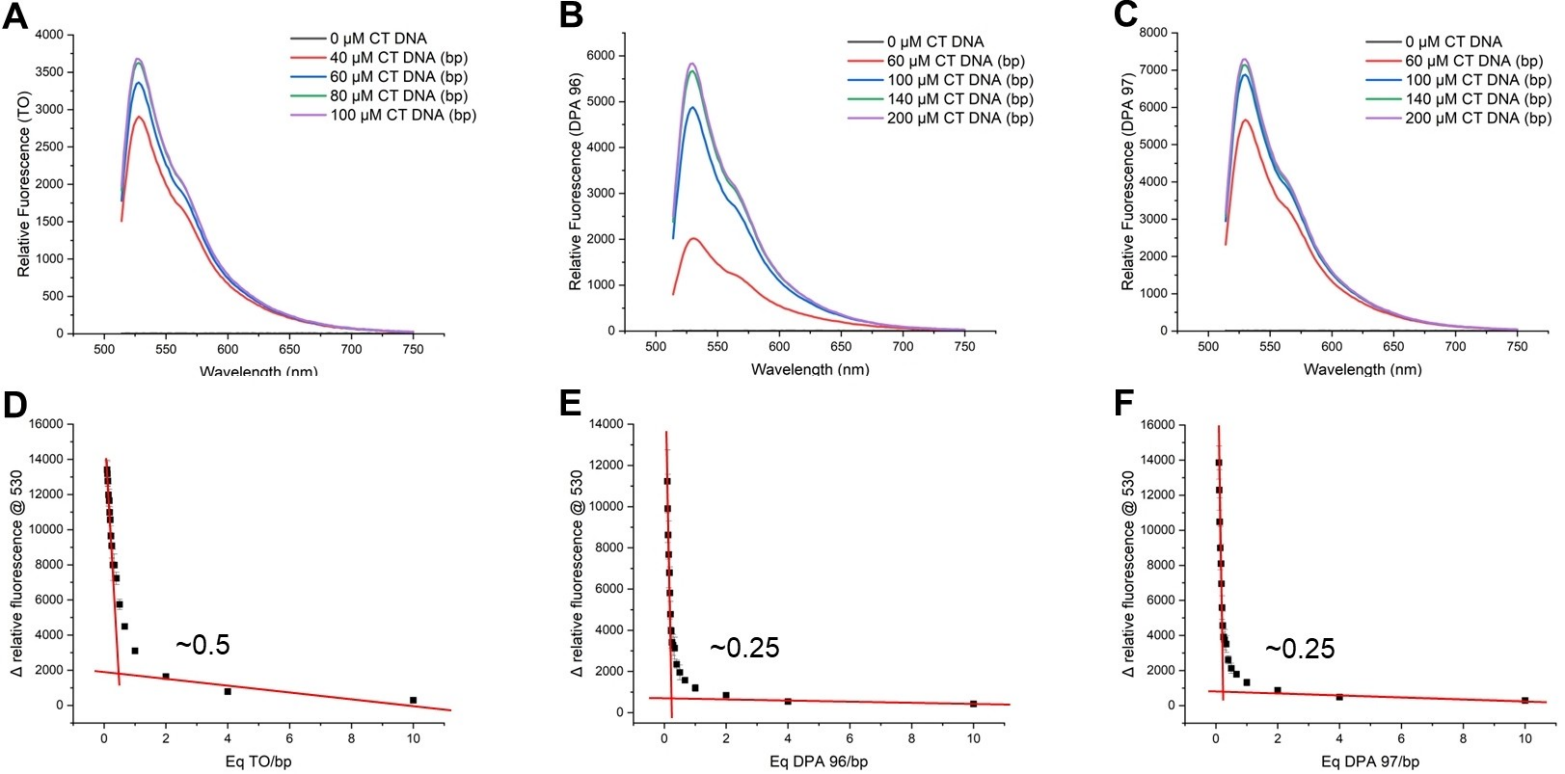
Relative fluorescence spectra of (**A**) TO, (**B**) **DPA96**, and (**C**) **DPA97** at a constant 5 μM with increasing concentrations of CT DNA (l_ex_/l_em_=488/514–750 nm); and saturation plots to determination of binding stoichiometry of (**D**) TO, (**E**) **DPA96**, and (**F**) **DPA97**.

The structure and synthetic scheme for TO‐NEO conjugates is given in Figure 1 and Scheme 1, respectively. The interactions of TO with CT DNA and other nucleic acids used in this study have been well characterized. Starting with calf thymus DNA (CT‐DNA), the relative fluorescence intensity spectra were obtained from samples containing a fixed concentration of TO, **DPA96**, or **DPA97** to which CT DNA was titrated. The relative fluorescence intensity spectra in Figure 2A–C show that the maximal emission wavelength was the same for TO‐NEO conjugates relative to the TO control.

However, there were significant differences in the concentration of CT DNA needed to deplete the free ligands from solution and reach peak fluorescence. TO had the lowest fluorescence, followed by **DPA96**, then **DPA97**. At the concentration of CT DNA that exhibited the highest fluorescence, **DPA96** was 1.6x brighter than TO, while DPA97 was 2x brighter. The need for a higher concentration of CT DNA to achieve saturation by TO‐NEO conjugates indicates that fewer molecules bind to the DNA as shown in Figure 2D–F that illustrates the binding stoichiometry of TO and the TO‐NEO compounds and CT DNA titrated saturation. Whereas ~0.5 TO molecules bind to CT DNA per base pair (as expected), only ~0.25 TO‐NEO molecules bind per base pair of CT DNA. We can conclude as expected, the structurally larger TO‐NEO conjugates span across a greater area of CT DNA binding at a average of 4 base pairs, as opposed to the well‐known binding of TO and other intercalators to every alternate base pair.

CT DNA is composed of approximately 42 % GC. As previously stated, NEO has a high affinity to GC‐rich sequences.^[15]^ Therefore, we investigated the binding characteristics of **DPA96** and **DPA97** with four short (8 base pair) GC‐rich sequences via comparison of relative fluorescence intensities.

Table 1 shows the change in relative fluorescence upon binding of TO‐NEO for DNA duplexes for Seq 1 (5’‐GGGGCCCC), Seq 2 (5’‐CCCCGGGG), GC tracts of the Sp1 (5’‐GGGGCGGG, Seq 3) and EGR1 (5’‐GCGGGGGC, Seq 4) transcription factors. The sequence 5’‐GGGGCCCC (Seq 1) was chosen for further investigation due to the high affinity of NEO for groove binding as observed in a previous study,^[15]^ thus providing an opportunity for the TO to intercalate or stack in the base pairs. The opposite sequence 5’‐CCCCGGGG (Seq 2) was used for a comparison. The specificity of **DPA96** and **DPA97** for Seq 1 did not follow the trend as expected for NEO binding. When comparing Seq 1 DNA and Seq 2 DNA duplexes, a larger increase in TO‐NEO relative fluorescence intensity was seen with Seq 2 DNA duplex instead. To report normalized data, full saturation must be achieved with all duplexes as the measurements are relative to each other. Fluorescence of the fully saturated duplexed of Seq 1–4 DNA, and ▵F values for **DPA96** and **DPA97** at a 1 : 1 concentration ratio with duplexed DNA varied greatly among the duplexes tested (Table 1).

**Table 1:** Relative ΔF values of duplexed DNA sequences 1–4 with the addition of **DPA96** and **DPA97**.^[a]^

Sequence	ΔF×10^3^ DPA96	ΔF×10^3^ DPA97
1 (GGGGCCCC)_2_	23.2	17.5
2 (CCCCGGGG)_2_	36.7	26.2
3 (GGGGCGGG)_2_	15.3	30.2
4 (GCGGGGGC)_2_	28.1	30.2

[a] The ratio of **DPA96** and **DPA97** to duplex DNA was 1 : 1. Abbreviations: ▵F: Relative change in fluorescence from background (~1400 relative fluorescent units).

To better understand how nucleic acid sequence may be impacting **DPA96** and **DPA97** binding interactions, a screening assay was performed with an additional 252 GC rich duplexes (Figures S1 and S2, Table S1). With respect to relative fluorescent signal for TO‐NEO, the top 20 and bottom 20 sequences were identified. Within the top 20, six sequences were shared between **DPA96** and **DPA97**; however, the ΔF values differed (Table S1). No correlation could be drawn that would predict which sequence would produce high relative fluorescence intensity with both compounds. Results between these two duplexes varied greatly, implying these conjugates display subtle differences in binding patterns based upon the sequence.

We then chose a sequence from Table S1 (5′‐GCGCCCGC, Seq 5) that yielded high ΔF values for both **DPA96** and **DPA97**, and does not form a G quadruplex under our soultion conditions. The sequence ranking was the second highest for **DPA96**, and sixth highest for **DPA97**. Additionally, a random sequence (5‘‐CTTCGATAGTG, Seq 6) with a lower GC percentage was used as a mixed base control.

### Competition Dialysis Studies for Relative Affinities, and Quantum Yields

**Figure 3:**
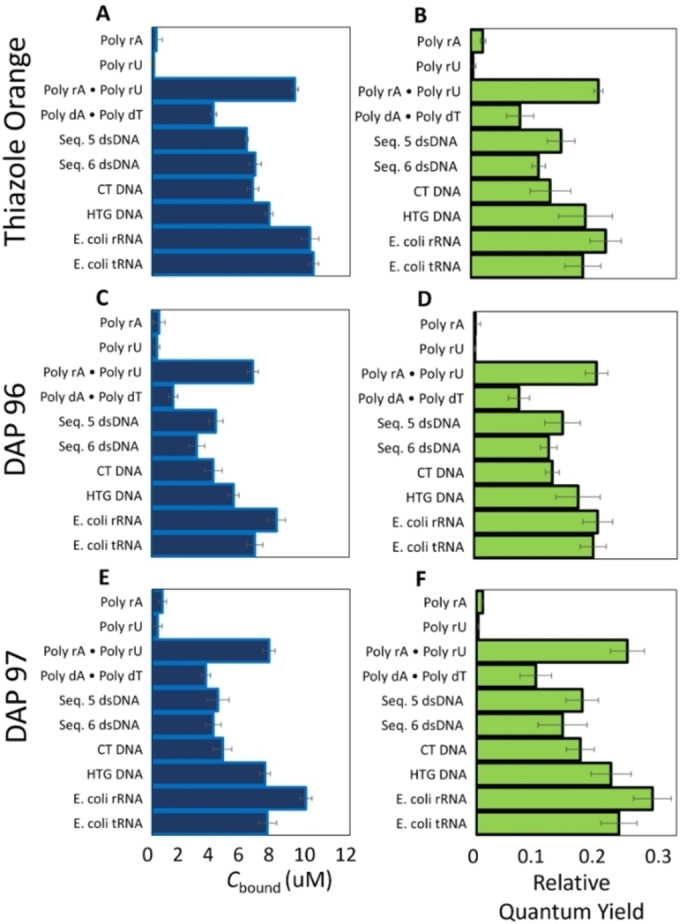
Competition dialysis data and relative quantum yields for TO (**A**, **B**), **DPA96** (**C**, **D**) and **DPA97** (**E**, **F**) for ligands bound to nucleic acids.

Competition dialysis was performed to determine the relative binding affinity of TO and TO‐NEO derivatives towards different nucleic acids (Figure 3A, C and E). A slightly higher affinity was observed with the GC rich DNA Seq 5 and Human Telomeric G‐quadruplex DNA (HTG DNA) as compared to the AT rich poly dA ⋅ Poly dT. RNA structures show even higher affinities for **DPA96** and **DPA97**.

Single point relative quantum yields obtained from the same solutions found the brightest signals for duplexed RNA (poly rA ⋅ poly rU), tRNA and rRNA, however there was little significant differences between TO, **DPA96** and **DPA97** using this method (Figure 3B, D and F). It should be noted that the single point measurement method is less accurate, providing relative quantum yields but the assay is amenable to a comparison of a large number of samples. Therefore, we investigated the relative quantum yields using the more rigorous and accurate method of titration of ligand into a fixed concentration of nucleic acid and then performing regression analysis for the integrated florescence as a function of absorbance. Relative quantum yields were calculated by this method similar to that used by Nygren et. al using the calculated concentrations of free and bound TO or DPA compound and the concentration of available binding sites.^[33]^ Fluorescein (FAM) was used as the reference standard as the absorbance and emission spectrum is similar to that of TO and the quantum yield is known for pH 7.0 used throughout these studies (Φ_FAM_: 0.75, neutral pH).[^34^, ^35^, ^36^] Similar absorbance and emission spectra for free FAM, free TO, **DPA96**, and **DPA97** bound to nucleic acids were observed (Figure S3). The relative fluorescence quantum yield was found for free TO and TO bound to CT DNA, Seq 5 DNA duplex, or Poly rA ⋅ Poly rU (Table 2). Relative to FAM (Φ_f_ 0.75, pH 7), free TO, **DPA96** and **DPA97** had a low fluorescence quantum yields Φ_f_ TO=1.5×10^−4^ which is in the range previously reported for TO and for **DPA96** Φ_f_=8.3×10^−4^ and **DPA97** Φ_f_=1.8×10^−4^.^[8]^ Compared to free TO, TO bound to calf thymus DNA and Seq 5 DNA duplex had ~850‐fold and 800‐fold higher fluorescence quantum yields, respectively (Φ_f_=0.122 and 0.116).

**Table 2:** Spectroscopic parameters and relative quantum yield for TO, **DPA96**, and **DPA97** associated with various nucleic acids sequences and structures.

Test Comparisons	ϵ_MAX_^[a]^ (M^−1^ cm^−1^)	λ_max(abs)_[nm]^[b]^	λ_max(em)_[nm]^[c]^	Φ_f_^[d]^, pH 7, 25 °C, SD
Free Dye FAM	70,000	488	517	0.75
Free Dye TO	61,128	504	NA	1.5×10^−4^
TO‐CT DNA	64,860	508	528	0.136
TO‐HTG DNA	73,000	514	528	0.129
TO‐Seq 5 dsDNA	74,540	508	528	0.121
TO‐Seq 6 dsDNA	64,860	508	528	0.137
TO‐Poly dA ⋅ Poly dT	ND	508	528	0.077
TO‐Poly rA ⋅ Poly rU	19,150	516	535	0.341
Free Dye **DPA96**	71,830	504	NA	8.3×10^−4^
**DPA96**‐CT DNA	74,650	512	528	0.209
**DPA96**‐HTG DNA	73,000	514	528	0.181
**DPA96**‐Seq 5 dsDNA	73,950	512	528	0.274
**DPA96**‐Seq 6 dsDNA	74,650	508	528	0.167
**DPA96**‐Poly dA ⋅ Poly dT	ND	508	528	0.089
**DPA96**‐Poly rA ⋅ Poly rU	19,150	516	535	0.317
Free Dye **DPA97**	66,040	504	NA	1.8×10^−4^
**DPA97**‐CT DNA	70,890	512	530	0.243
**DPA97**‐HTG DNA	73,000	512	530	0.224
**DPA97**‐Seq 5 dsDNA	71,760	512	530	0.252
**DPA97**‐Seq 6 dsDNA	70,890	508	530	0.194
**DPA97**‐Poly dA ⋅ Poly dT	ND	508	530	0.107
**DPA97**‐Poly rA ⋅ Poly rU	19,150	516	535	0.389

[a] The calculated extinction coefficient at pH 7. [b] Wavelength at the absorbance maximum, [c] Wavelength at the emission maximum, [d] Fluorescence quantum yield calculated from the regression plot of absorbance verses the integrated fluorescence using an excitation wavelength of 465 nm. Assays were performed in 10 mM HEPES pH 7, 100 mM KCl and 0.4 mM EDTA. The average of at least two replicates is given for quantum yield data. The absorbance spectra, fluorescence spectra and linear fits for this data is given in Figures S3–S6.

The quantum yields for HTG DNA were comparable to that of CT DNA and Seq 5 DNA duplex. Quantum yields were highest for poly(rA) ⋅ poly(rU) (TO, Φ_f_ 0.34), (**DPA96**, Φ_f_ 0.31) and (**DPA97**, Φ_f_ 0.38) with brightnesses that were ~2200‐fold higher, ~2000‐fold, and ~2500‐fold higher, respectively than that of free TO. **DPA96** and **DPA97** both demonstrated a ~1.5 to 2.5‐fold increase in quantum yield with CT DNA and the Seq 5 DNA duplex as compared to TO bound to CT DNA or the Seq 5 DNA duplex. Because the quantum yields of bound **DPA96** and **DPA97** were different, linker type between **DPA96** and **DPA97** may have influenced the binding modes and therefore brightness when bound to poly(rA) ⋅ poly(rU). The absorbance and fluorescence spectra for the titrations of TO, **DPA96**, and **DPA97** into CT DNA or duplexes and linear fits of the absorbance verses the integrated fluorescence is given in supplementary information (Figures S3–S6).

The ability of a ligand to stabilize nucleic acid can be reflected in the increase in thermal melting (T_m_) temperature^[37]^ and is expected to be a function of the ligand's binding affinity (Table 3, Figure S7). The increase in T_m_ due to the addition of TO was negligible for the Seq 5 RNA and Seq 5 DNA duplexes, with the highest increase seen in CT DNA at 1.5 °C as compared to the nucleic acid alone. **DPA96** and **DPA97** increased the T_m_ of CT DNA by 5.7 °C and 5.2 °C, respectively. It must be noted that these thermal denaturation studies were conducted at pre‐saturation levels to distinguish the effects of TO and TO‐NEO ligands. These low ratios (1 : 10::ligand:nucleic acid base pairs) allow for affinities of the tighter binding sites to be compared for the different ligands. A higher concentration of ligand was used for the HTG quadruplex, since the secondary structure formation here leads to a target G quadruplex structure whose final molarity is much lower than the single strand (per base composition).

**Table 3:** Comparison of the denaturation temperatures for duplexes, calf thymus DNA and HTG quadruplex.^[a]^

	Nucleic Acid (NA)	NA+TO	NA+DPA96	NA+DPA97
Seq 5 dsRNA	73±0.3	74±0.9	83±0.7	84±0.4
Poly rA ⋅ Poly rU	58±0.4	58±1.1	71±2.1	79±1.4
Seq 5 dsDNA	54±0.7	54±1.6	68±1.2	65±0.5
Seq 6 dsDNA	45±1.1	52±1.0	54±0.4	58±0.5
Poly dA ⋅ Poly dT	72±1.0	73±0.3	72±0.7	73±0.7
CT DNA	64±0.5	66±0.7	70±0.9	73±1.2
*HTG DNA	57±1.9	61±1.2	65±1.5	67±1.2
**HTG DNA	57±0.9	62±1.3	67±0.7	70±1.6

[a] Thermal melting assays were performed in 10 mM HEPES buffer with 0.4 mM EDTA and 100 mM KCl for all nucleic acids except for CT DNA. CT DNA melting curves were done in 10 mM cacodylate with no additional salts. The concentration of the nucleic acid duplexes was 5 μM. Ligands were at a 1 : 10 ratio with nucleic acid (per base pair). *For the Quadruplex secondary structure: A 2.2 to 1 ratio of ligand to HGT DNA was also used to allow stacking on both faces of the HTG. **Saturation of HTG using an even higher concentration of ligand (22 to 1) does not lead to much further changes in T_m_. The standard deviation (±) of the mean of three replicates on separate occasions is given.

A much greater increase was seen with the Seq 5 RNA, Seq 5 DNA duplexes, HTG DNA and Poly rA ⋅ Poly rU. The T_m_s for Seq 5 RNA duplex increased by 9.7 °C with **DPA96**, and 11 °C when **DPA97** was added to the sample. Seq 5 DNA duplex T_m_s were increased by 13.8 °C and 10.8 °C for **DPA96** and **DPA97**, respectively. Similar increases in T_m_ were observed for **DPA96** and **DPA97** bound HTG DNA with a smaller increase in T_m_ observed for TO bound HTG DNA. A direct comparison of **DPA96** and **DPA97** bound Poly rA ⋅ Poly rU and Poly dA ⋅ Poly dT duplex stabilization revealed that the RNA duplex is stabilized to a far greater extent than the DNA duplex. Thermal melting data suggest that the TO‐NEO compounds effectively bind to GC rich DNA, RNA structures, and HTG DNA but do not bind well to AT rich DNA, corroborating the competition dialysis and quantum yield data.

### Cellular Uptake

Cell culture studies were conducted to evaluate the intracellular distribution of TO and the TO‐NEO derivatives. TO readily enters eukaryotic cells; however, NEO shows little to no cellular uptake. In order to be a useful stain, the TO‐NEO conjugates need to be able to permeate cells. Uptake studies done with various cell types and the TO‐NEO conjugate compounds reveal that when bound to TO, NEO is able to enter the mammalian cells.

**Figure 4:**
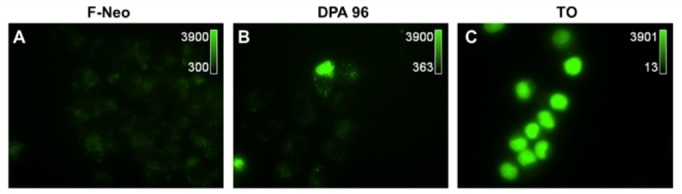
Epifluorescence microscopy images of J774 A.1 macrophage cells after 24 hours of treatment with(**A**) F‐NEO, (**B**) **DPA96** or, (**C**) TO.

A comparison of TO‐NEO with F‐NEO (a fluorescein linked NEO created in our laboratory^[38]^ was performed in mammalian cells. Cellular uptake was characterized by treating cells for 24 hours with 5 μM of a fluorescein‐NEO conjugate (F‐NEO) TO‐NEO conjugate **DPA96**, or TO. After treatment, a strong fluorescent signal was observed from **DPA96** and TO, but not for the F‐NEO conjugate. Fluorescent microscopy of treated J774 A.1 cells indicates that the TO moiety was responsible for cellular uptake and not the NEO moiety because NEO conjugated to fluorescein was unable to effectively enter the cells (Figures 4 and S8). Since F‐NEO is highly fluorescent, microscopy images reveal that fluorescein alone will not help facilitate uptake of NEO. F‐NEO treated cells were also imaged without standardizing the microscope parameters to the TO settings in order to confirm low cellular uptake (Figure S8B). However, little to no relative fluorescence was detected beyond background or autofluorescence, thus confirming our previous conclusion.

Other cell lines were tested as well which included DU‐145, MCF‐7, and HEK‐293 cells analyzed via fluorescence microscopy and flow cytometry. Flow cytometry confirmed uptake of the DPA compounds. The microscopy visually confirmed that all TO compounds readily entered each cell type, however only the DPA compounds exhibited a more defined localization of the TO‐NEO conjugates (data not shown).

We investigated a more refined localization using MCF‐7 and J774 A.1. The larger size of these cell types, provided us with a better differentiation between organelles. A shorter time of exposure was also used so that the cells were not fully saturated with TO or TO‐NEO, thus allowing us to better see the localization of these compounds. To rule out localization of the TO‐NEO compounds being attributed to binding to actin fibers of the cell cytoskeleton or the interaction of cationic dyes with a negatively‐charged mitochondrial membrane, additional co‐stains were used which included: Hoechst 33342 to show DNA within the nucleus, phalloidin to visualize the actin fibers of the cell cytoskeleton, or mitochondria‐associated staining with MitoBright LT.

**Figure 5:**
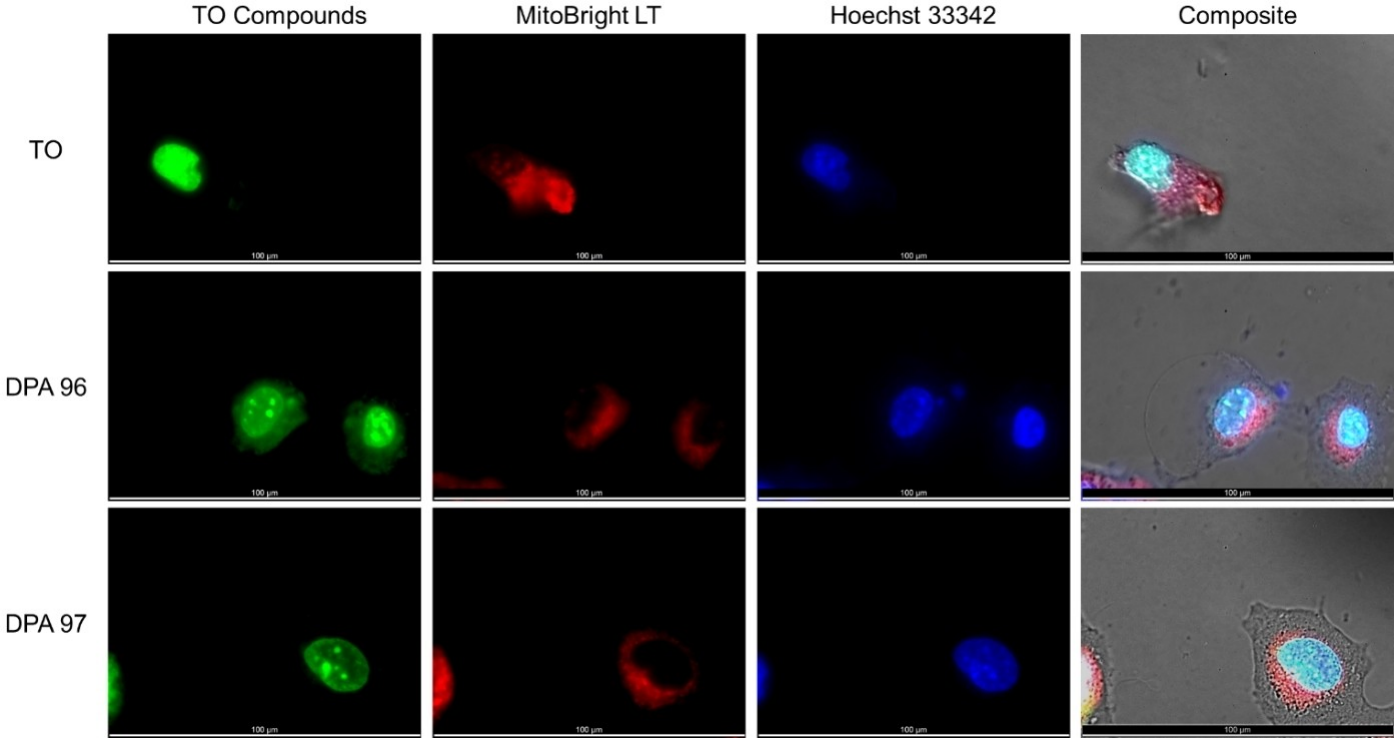
Co‐staining of living MCF7 cells with TO, **DPA96**, or **DPA97** (0.5 μM), Hoechst 33342 and mitochondria‐staining probe (MitoBrigtht LT Deep Red). The cells stained with TO compounds (20 min) were further incubated in complete media containing co‐staining probe for 20 min. After washing with D‐PBS buffer twice, the cells were imaged in D‐PBS buffer.

Alexa Fluor 594 phalloidin staining after treatment of macrophage cells with **DPA96** or **DPA97** confirms that the compounds are not localized to the actin fibers (Figure S9). The phalloidin staining clearly shows minimal TO‐NEO associated fluorescence detected in the red fluorescence region associated with phalloidin. Hoechst 33342, MitoBright LT Deep Red, and the TO compound co‐stained cells are shown Figure 5. There is little co‐localization of TO‐NEO (green) and mitochondria (red) which are localized to the cytosol outside the nucleus. However, some overlap is observed and may be due to lower uptake in mitochondria and the affinity of the conjugated NEO to mitochondrial RNA along with intercalation of TO to mitochondrial DNA. The co‐staining allowed us to differentiate localization of TO‐NEO to the nucleoli of the nucleus from that of the mitochondria or actin fibers. We see the similar mitochondrial staining pattern in J774.A1 macrophage cells (Figure S10).

**Figure 6:**
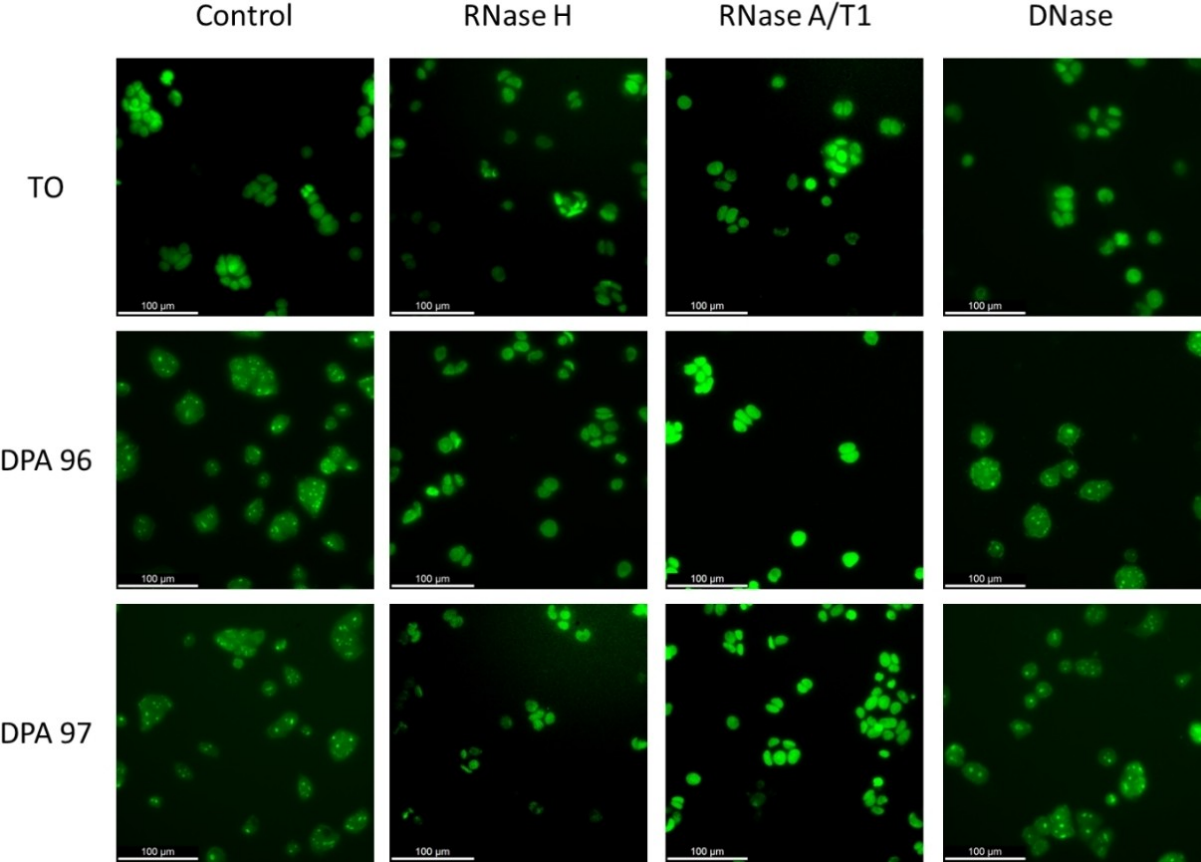
MCF‐7 cells were fixed in pre‐chilled methanol at −20 °C for 1 min. The cell membrane was then permeabilized with 1 % Triton X‐100 in D‐PBS for 2 min at room temperature. After rinsing with DPBS twice, the cells were incubated with 1.0 μM TO, **DPA96**, or **DPA97** in D‐PBS solution for 20 min at 37 °C in a 5 % CO_2_ atmosphere, followed by washing with D‐PBS buffer twice. DNase (100 U/mL), RNase A/T1 (100 U/mL), or D‐PBS (control) were added into each of the three cells, which was then incubated at 37 °C in a 5 % CO_2_ atmosphere for 3 h. Cells were rinsed again by D‐PBS buffer twice before imaging. The fluorescent imaging pictures were obtained in D‐PBS buffer by using an equal exposure time for control, DNase, and RNase experiments.

MCF‐7 cells were used to determine if localization resulted from binding to RNA or DNA in cells treated with DNase, RNase A/T, or RNase H. Figure 6 shows localization in the cells treated with **DPA96** and **DPA97**, with TO. Both TO‐NEO conjugates are fluorescent inside the cells, and exhibit more localization than TO.

Additional studies were performed to determine which type of nucleic acid the TO‐NEO associated fluorescence was being emitted.

To investigate whether the localization correlated for double stranded DNA⋅DNA or RNA⋅RNA or RNA⋅DNA hybrids, cells were treated with RNase H which eliminates RNA⋅DNA hybrids, RNase A/T1 which is a mixture that eliminates dsRNA, or DNase which eliminates DNA.[^39^, ^40^] The affinity of the TO‐NEO for RNA was evident according to microscopy experiments. Treating the cells with either RNase A/T or RNase H resulted in the loss of the localization of TO‐NEO fluorescence. The experiments involving fixed cells exposed to the TO compounds followed by treatment with RNase A/T, RNase H, and DNase were repeated with additional cell lines (Figure S11) with the same resulting localization. Neomycin binding to rRNA (biological target of aminoglycosides) can explain the nucleolus specific binding of **DPA96** and **DPA97**.

### Cytotoxicity of TO‐NEO Conjugates

**Figure 7:**
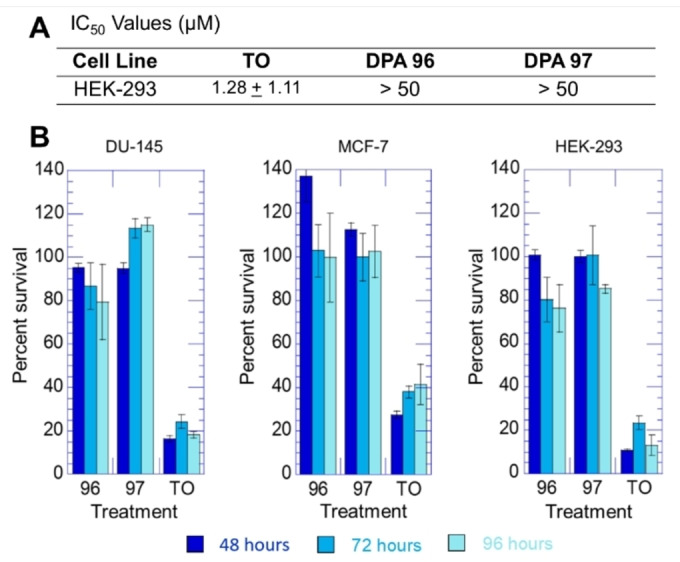
(**A**) 24‐hour IC_50_ values (μM) calculated for TO and TO‐NEO conjugates in kidney cell line, HEK‐293. (**B**) Time course study of cell viability with uptake of TO‐NEO conjugate compounds and TO. Each of the cell lines were plated in a 96 well plate. 10 μM each of the compound for 48; 72; and 96 hours the cell viability was assayed with CCK‐8 reagent by reading the OD at 450 nm as described under materials and methods.

HEK‐293 cells were chosen as a representative cell line to evaluate toxicity. The high IC_50_ values for **DPA96** and **DPA97** as compared to TO (Figure 7A) indicated significantly low cytotoxicity for the TO‐NEO conjugate compounds as compared with TO. **DPA96** and **DPA97** showed little toxicity at concentrations as high as 50 μM. The toxicity of TO may be due to its ability to bind with regulatory regions of DNA, leading to growth arrest. It is well documented that the small molecule interaction with gene promoter regions leads to aberrant gene transcription as well as binding to DNA replication/repair sites leading to cell death.^[11]^

Cellular toxicity of TO, **DPA96**, and **DPA97** using the Cell Counting Kit‐8 (CCK‐8) assay that measures cellular metabolic rate in a time course dose dependent study was completed using the cell lines DU‐145, MCF7, and HEK‐293. We found that TO alone was highly toxic with more than 80 %, 70 %, 90 % of the cells killed at 48 hours of treatment with a concentration of 10 μM, respectively. All TO‐NEO conjugates showed less toxicity as compared with TO alone, reported in Figure 7B.

In summary, several independent experiments were conducted to obtain binding and fluorescence characteristics of two TO‐NEO conjugates. We demonstrate that conjugation of NEO and TO significantly improves the stability of RNA duplexes as compared to unmodified TO, and there appears to be a higher degree of GC rich DNA sequence selectivity as well. Further studies are warranted to further elucidate the trend in sequence and structure selectivity, to identify the high affinity sites in the genome for further detailed biophysical investigations. Fluorescence microscopy images of mammalian cells revealed that TO‐NEO localizes to the nucleoli, likely binding to nascent rRNA, within the nucleus as compared to a more diffuse fluorescence observed with TO in the nucleus. Additionally, an improvement in uptake of NEO, while also decreasing the cytotoxicity of TO in eukaryotic cells was observed. Conjugated TO‐NEO can be used as a less cytotoxic alternative for cellular labeling for both flow cytometry and fluorescence microscopy. In conclusion, the data showed that the conjugation of TO and NEO improved some of the adverse properties of each compound alone. NEO, which was previously unable to enter eukaryotic cells without a vehicle was imaged in mammalian cells. The cytotoxicity of TO was improved when conjugated to NEO. Furthermore, our findings suggest that the linker length plays a role in relative fluorescence intensity depending on the DNA sequence, and more linker lengths should be investigated for optimization of the probes for specific sequences. Further studies need to be conducted to determine the sequence dependence and exact site of localization of the TO‐NEO compounds.

## Methods

### Reagents and Nucleic Acids

DNA duplexes (5′‐GCGCCCGC, Seq 5 and 5‘‐CTTCGATAGTG, Seq 6) and human telomeric G‐quadruplex (HTG, d[AG_3_(T_2_AG_3_)_3_] were purchased as solids from Integrated DNA Technologies. Poly dA ⋅ poly dT, poly rA ⋅ poly rU, rRNA and tRNA were purchased as solids from Sigma Aldrich. Calf thymus was purchased from Fisher Scientific as a solution (10 mg/mL).

### Relative Fluorescence Intensity of TO and TO‐NEO Conjugates Associated with Nucleic Acids

Relative fluorescence intensity was recorded for TO and the TO‐NEO conjugates (5 μM) in buffer containing 50 mM NaCl, 10 mM sodium cacodylate, 0.5 mM EDTA, pH 6.0 using a plate reader (Tecan Genios Pro). Fluorometer parameters were consistent for all samples, λ_ex_/λ_em_=488 nm/514–750 nm. CT DNA was titrated into TO or TO‐NEO solutions and fluorescence scans identified the optimum concentration of CT DNA for the brightest signal from each compound. The maximum relative fluorescence intensity was then used to determine the relative amount of compound in each cell for microscopy and flow cytometry studies (due to excess cellular genomic DNA relative to the drug concentration).

### Relative Fluorescence Intensity of TO and TO‐NEO Conjugates for 256 Eight Base‐Pair Sequences

DNA purchased from IDT or Operon was dissolved in DNase free water and the concentrations were determined using UV absorbance of the single strands at 90 °C and the extinction coefficients provided by IDT or Operon. DNA duplexes were formed using a water bath to heat the sample to 90 °C for ~5 minutes, allowed to cool to room temperature, then stored at 4 °C until used in the various experiments. DNA concentration was 1 μM/duplex in 50 mM NaCl, 10 mM SC, 0.5 mM EDTA (pH 6.0). **DPA96** or **DPA97** was added at a 1 : 1 concentration ratio with the duplexed DNA. DNA was added to buffer in the 96 well plates and incubated for 24 hours at 4 °C prior to running the assay on the plate reader.

### Binding Stoichiometry

Using a plate reader (Tecan SPARK) and a black walled 96‐well plate, CT DNA was titrated into TO or TO NEO by adding 100 μL of 2 μM of ligand to 100 μL of the appropriate concentration of CT DNA in each well. The assay buffer consisted of 10 mM HEPES (pH 7), 0.4 mM EDTA, 100 mM KCl. Wells containing only buffer, buffer and TO or TO‐NEO compound, and buffer with CT DNA were used for baseline corrections. At each CT DNA concentration, a control without TO or TO‐NEO was used for background subtraction. Fluorescence emission spectra were collected from 514 to 750 nm, with an excitation wavelength of 488 nm. Relative fluorescence intensity data is plotted as the fraction of the fluorescence maximum per plate. Determination of binding stoichiometry was done from analysis of saturation plots using ORIGIN PRO graphing software.

### Relative Quantum Yield

The microtiter plate method was used for determination of quantum yields for **DPA96** and **DPA97** relative to fluoresceine (FAM) for which the quantum yield is known (Φ=0.9–1, alkaline pH, 0.75 neutral pH). All experiments were performed in the same buffer: 10 mM HEPES (pH 7.0), 100 mM KCl, 0.4 mM EDTA in 96 well clear bottom black walled plates at 25 °C, with a final volume of 100 μL for each sample and sample dilution. Duplex nucleic acid concentrations were 1 μM and the concentration range for FAM, TO, **DPA96**, and **DPA97** was 0.25–16 μM. All sample absorbances were adjusted to a maximum absorbance of 0.100 AU for the most concentrated dilution and then serially diluted in buffer to an absorbance of 0.03. Absorbance and fluorescence scans were taken using a TECAN SPARK plate reader quipped with Magellan software for spectral reduction and background subtraction of the integrated areas for the fluorescence scans (emission 480–700 nm). An absorbance scan of samples (340–700 nm) identified the absorbance overlap region and excitation wavelength to be used for fluorescence scans (excitation at 465 nm). Plate parameters for acquisition of fluorescence data were: excitation: 465 nm with a bandwidth 5 nm, emission range: 480–710 nm with a bandwidth 5 nm, step size 1 nm, 30 flashes/well, settle time 50 ms, and integration time 40 us. The fluorescence optimal gain was determined on the most concentrated dilution samples (calculated from most concentrated FAM solution well). Spectral correction for fluorescence scans (background subtraction, smoothing, integration 465–700 nm was performed using Magellan software for the TECAN SPARK plate reader. Raw data exported and linear regression analysis of the plot of absorbance vs integrated fluorescence intensity was performed using ORIGIN PRO software. The resulting slope of the line was used to calculate the relative quantum yield using the following equation:

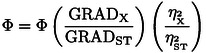




Where subscripts ST and X denote the standard and test sample, respectively Φf is the fluorescence quantum yield, Grad is the gradient from the plot of the integrated fluorescence intensity vs absorbance and quantum yield for the test sample, Φ_ST_ is the quantum yield for the reference standard fluorescein (0.75, neutral pH), GradX is the integrated fluorescence vs absorbance and η is the refractive index of the solvent. The same buffer was used for the reference standard fluorescein as for the test samples thus the refractive index factor cancels to 1.

### Competition Dialysis: Binding and Single Point Quantum Yields

The protocol for the competition dialysis assay was followed as reported.^[41]^ Nucleic acid solutions were prepared with phosphate buffer (6 mM Na_2_HPO_4_, 2 mM NaH_2_PO_4_; pH 7.0) with 185 mM NaCl and 1 mM Na_2_EDTA to prepare 75 μM (monomeric unit). The nucleic acid solution (200 μL) was placed into mini dialysis units and allowed to equilibrate against 400 mL phosphate buffer containing 1 μM thiazole orange, **DPA96** or **DPA97** at room temperature for 24 h with continuous stirring. At the end of the equilibration period, 180 μL of the nucleic acid sample was removed and deposited in a black walled 96 well clear bottom plate and the absorbance spectra (340–700 nm) and fluorescence spectra 480–700 nm emission with 465 nm excitation were collected using a plate reader. The spectral correction and integrated fluorescence spectra were collected using Magellan software for the TECAN SPARK plate reader. After collection of absorbance and fluorescence spectra, each sample well received 20 μL of a 10 % sodium dodecyl sulfate (SDS) solution (w/v) added at a final concentration of 1 %. The plate was allowed to incubate for 15 minutes before determining the ligand concentration spectrophotometrically. Absorbance spectra were checked to confirm denaturation of the nucleic acids and disassociation of free ligand. The correction to the absorbance reading was made by multiplying by a factor of 1.11 to account for dilution of sample from addition of SDS. The data from the competition dialysis was used to calculate the apparent binding constant (K_app_) as follows:

$$K_{app}=\left(\frac{C_{b}}{C_{f}}\right)\left(S_{total}-C_{b}\right)$$

where C_b_ is the concentration of bound ligand, C_f_ is the concentration of the free ligand (1 μM in this study) and S_total_ is the total nucleic acid concentration (75 μM, monomeric unit). These ligand‐bound nucleic acid samples were also assayed for relative quantum yield as described in the methods section.

### Cell Lines

MCF‐7 cell line was a gift from Dr. Chen from Clemson University (Clemson, SC). J774 A.1 macrophage, DU‐145, and HEK‐293 cell line was purchased from ATTC. The other chemicals were of the highest purity commercially available through Sigma‐Aldrich.

### Cell Culture

MCF‐7 and DU‐145 cells were grown in RPMI medium, whereas HEK‐293 cells were grown in DMEM (Dulbecco's Modified Eagles Medium) in a humidified incubator at 37 °C supplied with 5 % CO_2_. Both culture media were supplemented with 10 % heat inactivated Fetal Bovine Serum (FBS), Penicillin (100 units/mL); Streptomycin (100 μg/mL); and L‐glutamine (2 mM). The viability of the cells was 97 % or more with each passage, as determined by Trypan blue exclusion assay.

### Microscopy: Compound Uptake

Cells were grown on 15 mm circular cover glasses placed in 24 well plastic tissue culture plates until the cells occupied around 80 % of the culture plates, at which time the cells were incubated with 5 μM compounds as indicated in the figure legend for 16 hours, washed three times with Ringer's Buffer (RB; 10 mM HEPES, 155 mM NaCl, 5 mM KCl, 2 mM CaCl_2_, 1 mM MgCl_2_, 2 mM NaH_2_PO_4_, 10 mM glucose, pH 7.4), and chased for 4 hours prior to imaging. Relative fluorescence intensity imaging was performed by inverted epifluorescence microscope (Olympus IX71, 60 X/1.45 N.A. objectives with xenon arc lamp as excitation source). The cells were fixed with 4 % paraformaldehyde solution and the cover glasses were mounted on glass slides. The cellular uptake of these compounds was viewed with 494 nm excitation (20 nm bandpass) and 531 nm emission (22 nm bandpass) as is characteristic for TO relative fluorescence intensity.

### Flow Cytometry: Compound Uptake

Cells grown in 35 mm culture dishes were incubated with each of the compounds to be tested separately in culture medium as described earlier. The incubation was for 8 to 10 hours at a concentration of 5 μM for each compound. At the end of each incubation the cells were detached from the bottom of the plate by mild trypsinization. Detached cells were pelleted, washed three times with 1x phosphate buffered saline (PBS), and suspended in 1x PBS. 10,000 cells were screened by flow cytometer (BD FACS Calibur) at excitation and emission wavelengths of 488 nm and 530 nm, respectively. The data from the flow cytometer was analyzed using FlowCell Quest as detailed in Fernando et al. 2010.^[7]^

### Cytotoxicity

Cells from each cell line were seeded at 15,000 cells per well in a 96 well plate and allowed to attach to the well surface. After cells had attached to the well surface, compound was added to the appropriate wells to a final concentration of 10 μM and incubated according to cell culture conditions for 48, 72, or 96 hours. At the indicated time (48; 72; 96 hours) 10 μL of CCK‐8 reagent was added to each well. The optical density (OD) at 450 nm of each well was read using a plate reader (Tecan Genios Pro) at 3 hours after addition of the CCK‐8 reagent, as described by Dojindo kit instructions. The mean OD of 4 wells per each drug/time point after subtracting the background OD value was determined by plotting a ratio of non‐treated controls at each of the indicated time points.

### IC_50_ Cytotoxicity Studies

The IC_50_ cytotoxicity study was completed similarly to the method described by Rebibo‐Sabbah et al.^[42]^ HEK‐293 cells were seeded at 10,000 cells per well in a 96 well plate and returned to culture conditions overnight. The next day, each test compound was diluted to different concentrations in culture media without Pen/Strep. Each concentration of compound was tested in triplicate and added to the prepared cells. Compounds TO, **DPA96**, and **DPA97** were tested up to 50 μM. Cells were incubated with treatments according to cell culture conditions for 48 hours. After 48 hours, 10 μL of CCK‐8 reagent was added to each well. The optical density (OD) at 450 nm of each well was read using a plate reader (Tecan Genios Pro) 3 hours after addition of the CCK‐8 reagent, as described by Dojindo kit instructions. The cell viability was calculated as a ratio of the number of live cells in the wells treated versus the number of live cells present in the untreated wells. The IC_50_ was determined by fitting the curve of the normalized cell viability versus the log of the drug concentration using Origin 5.0 software.

### Compound Synthesis and Characterization

The full **DPA96** synthesis scheme and characterization can be found in Figure S12. **DPA97** was synthesized as previously reported.^[30]^

## Supplementary Information

Supplementary information includes characterization of ΔF for complete list of 8 bp GC rich DNA sequences with TO‐NEO compounds. Absorbance and fluorescence scans used for quantum yields. Linear fitted data for determing quantum yields. Single point quantum yields. Thermal melting curves. Relative fluorescence intensity microscopy images of Alexa Fluor 594, MitoTracker Red, and Hoechst 33258 stained macrophages following treatment with TO‐NEO compounds are also included. Full synthesis scheme and characterization for **DPA96**.

## Conflict of Interests

DPA has ownership interest in NUBAD LLC.

1

## Supporting information

As a service to our authors and readers, this journal provides supporting information supplied by the authors. Such materials are peer reviewed and may be re‐organized for online delivery, but are not copy‐edited or typeset. Technical support issues arising from supporting information (other than missing files) should be addressed to the authors.

Supporting Information

## Data Availability

The data that support the findings of this study are available in the supplementary material of this article.
