# Supplementary material for: Nucleic Acid Specificity, Cellular Localization and Reduced Toxicities of Thiazole Orange‐Neomycin Conjugates
Source: ChemistryOpen. 2024 Dec 27;14(4):e202400189. doi: 10.1002/open.202400189 (PMC11973503; doi:10.1002/open.202400189)
Supplement: Supplementary file 1 — Supporting Information [file OPEN-14-e202400189-s001.pdf]

ChemistryOpen

Supporting Information

Nucleic Acid Specificity, Cellular Localization and Reduced Toxicities of Thiazole Orange-Neomycin Conjugates

Antwine W. McFarland, Jr., Lawrence P. Fernando, Patrick Kellish, Sandra P. Story, Gretchen B. Schober, Sunil Kumar, Changjun Gong, Ada King, Xianchang Gong, Alain S. Leutou, and Dev P. Arya*

Supporting Information:

Nucleic Acid Specificity, Cellular Localization and Reduced Toxicities of Thiazole Orange-Neomycin Conjugates

Antwine W. McFarland Jr.[1], Lawrence P. Fernando [1], Patrick Kellish[2], Sandra P. Story[1], Gretchen B. Schober[1], Sunil Kumar[2], Changjun Gong[2], Ada King[1], Xianchang Gong[1], Alain Leutou[1], Dev P. Arya*[1,2]

[1]NUBAD LLC, Greer, 29650, USA

[2]Department of Chemistry, Clemson University, Clemson, SC 29634 (USA)

** Correspondence and requests for materials should be addressed to D.P.A. (email: dparya@clemson.edu)*
Phone [1](864) 455-1463; [2](864) 656 1106
Fax [2](864) 656 6613

Supporting Information Includes:

Figures S1-2:	Plots depicting change in fluorescence of DPA 96 and DPA 97 in combination with GC rich 8 bp DNA sequences.
Table S1:	Change in fluorescence values for top 20 DNA duplexes with TO-Neo compounds.
Figure S3:	Combined absorbance and fluorescence emission spectra of fluorescein, thiazole orange, DPA96, and DPA97 into calf thymus DNA, Seq 5 dsDNA, or poly (rA) ● poly (rU).
Figure S4:	Absorbance scans for titration of fluorescein, thiazole orange, DPA96, and DPA97 into calf thymus DNA, Seq 5 dsDNA, or poly (rA) ● poly (rU), Poly(dA) ● Poly(dT), and Seq 6 dsDNA.
Figure S5:	Fluorescence scans for titration of fluorescein, thiazole orange, DPA96, and DPA97 into calf thymus DNA, Seq 5 dsDNA, or poly (rA) ● poly (rU), Poly(dA) ● Poly(dT), and Seq 6 dsDNA.
Figure S6:	Linear fitted data plots of the integrated fluorescence intensity as a function of absorbance over the titration range of thiazole orange, DPA 96, and DPA 97 into calf thymus DNA, Seq 5 dsDNA, or poly (rA) ● poly (rU), Poly(dA) ● Poly(dT), and Seq 6 dsDNA.
Figure S7:	Thermal melting curves Seq 5 dsRNA, Poly rA ● Poly rU, Seq 5 dsDNA, Seq 6 dsDNA, Poly dA ● Poly dT, and CT DNA without or in the presence of TO, DPA 96, or DPA 97.
Figure S8:	Structure of F-Neo and epifluorescence microscopy image of J774A.1 macrophage cells after 24 hours of treatment with F-Neo.

Figure S9-10:	Fluorescence microscopy of Alexa Fluor 594, MitoTracker Red, and Hoechst 33258 stained macrophages following treatment with TO-Neo compounds.
Figure S11:	RNase and DNase treated NIH-3T3 cells after staining with TO, DPA 96, or DPA 97.
Figure S12-S17:	Synthesis and characterization of DPA 96.

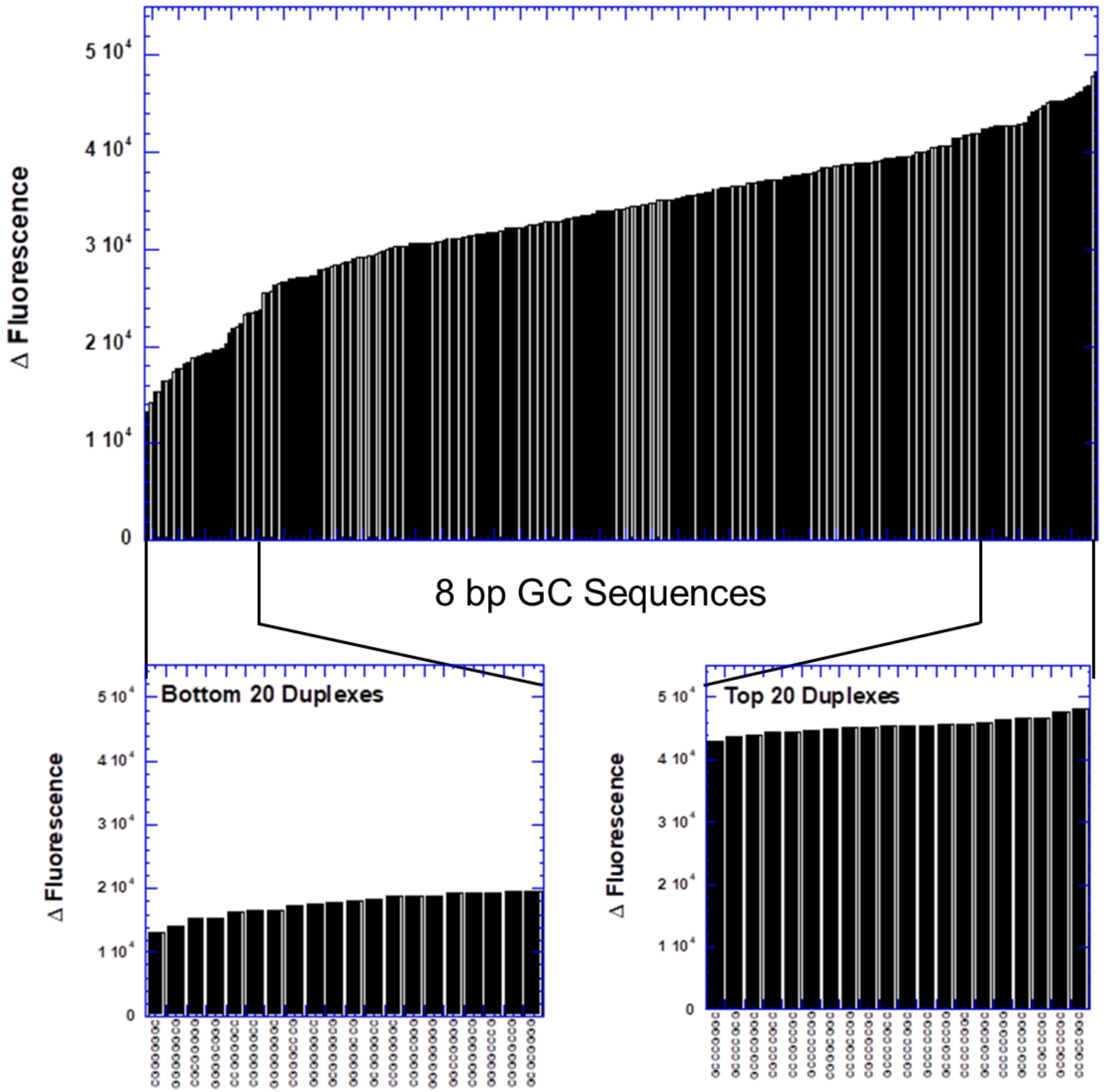

Figure S1. Plots depicting change in fluorescence of DPA 96 in combination with GC rich 8 bp DNA sequences. Offset plots focus on the bottom 20 sequences, which produce the lowest ΔF, and the top 20 sequences, which produce the largest ΔF.

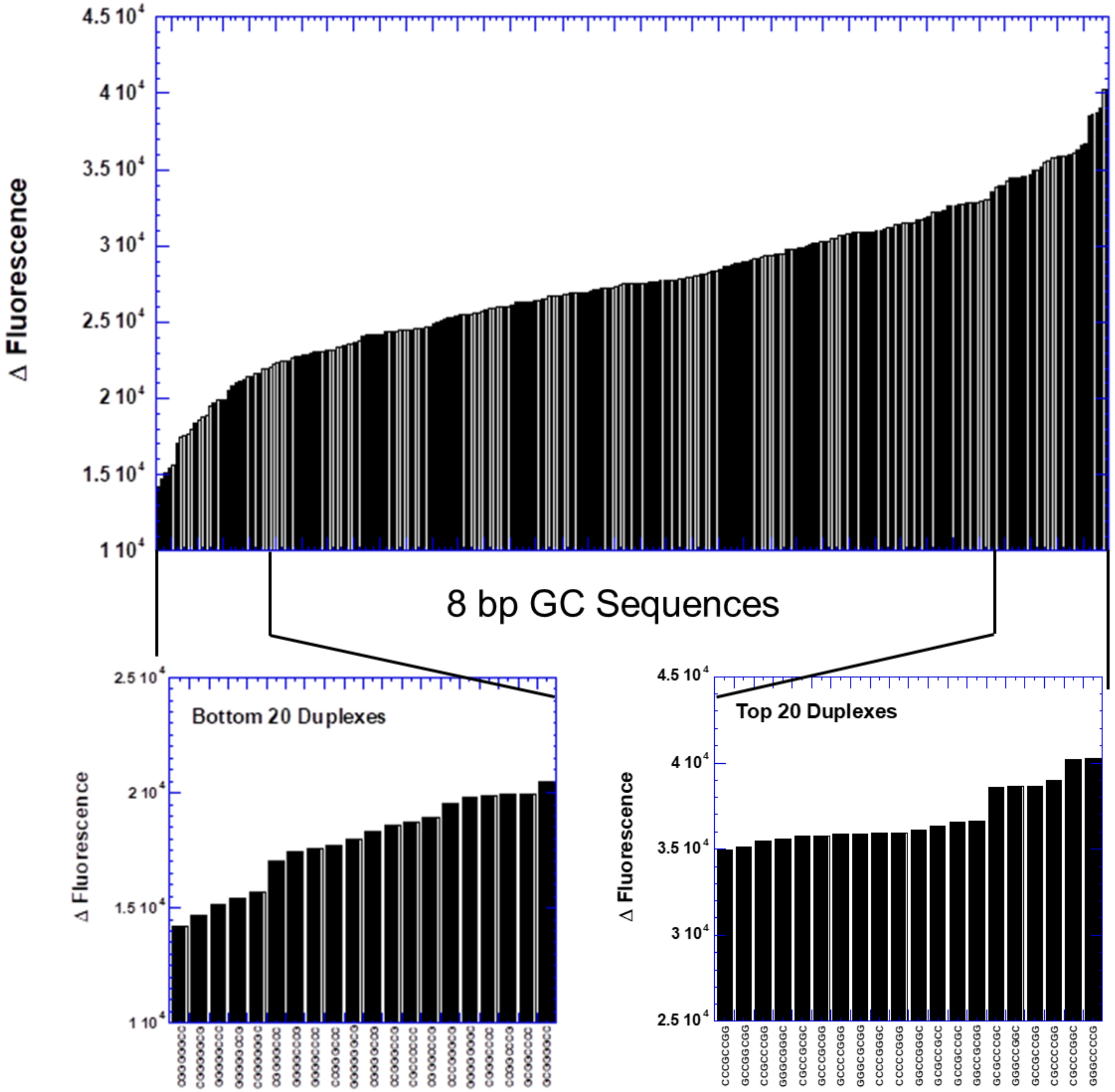

Figure S2. Plots depicting change in fluorescence of DPA 97 in combination with GC rich 8 bp DNA sequences. Offset plots focus on the bottom 20 sequences, which produce the lowest ΔF, and the top 20 sequences, which produce the largest ΔF.

Table S1. Change in fluorescence values for the top 20 duplexes with the addition of 1 r_{dd} (**A**) DPA 96 or (**B**) DPA 97. DNA concentration was 1μM/ duplex in 50 mM NaCl, 10 mM SC, 0.5 mM EDTA (pH 6.0). The DNA duplexes were added to buffer in the 96 well plates and incubated for 24 hours at 4°C prior to running the assay on the plate reader.

DAP96

Rank	Core Sequence	Δ Fluorescence with 1:1 r_{dd}
1	5'-CCGCCGGC	48268
2	**5'-GCGCCCGC**	**47779**
3	5'-CCGCCCGC	46808
4	5'-GCGCCGGC	46677
5	5'-CCCCCGGG	46320
6	5'-GCCGCGGC	46079
7	5'-CCCCCGGC	45739
8	5'-GCCGCCGG	45631
9	5'-GCCCCCGC	45458
10	5'-CGCCCGGC	45317
11	5'-GCCCCGCC	45317
12	5'-CGCCGCGC	45237
13	5'-CCCGCCGG	45191
14	5'-CGGGCGGC	45032
15	5'-GCCCCCGG	44825
16	5'-CCCGCCGC	44417
17	5'-CGCCCGCC	44360
18	5'-GCGCGGGC	44129
19	5'-GCCCCGCG	43614
20	5'-GCCCGGGC	43011

A

DPA97

Rank	Core Sequence	Δ Fluorescence with 1:1 r_{dd}
1	5'-GGGCCCCG	40270
2	5'-CGCCCGGC	40208
3	5'-CGCCCCGG	38985
4	5'-GCCGCCGG	38671
5	5'-GGGCCGGC	38628
6	**5'-GCGCCCGC**	38564
7	5'-GGCCGCGG	36627
8	5'-GCCGCCGC	36584
9	5'-GCGCCGCC	36306
10	5'-GGCCGGGC	36086
11	5'-CCCCCGGG	35965
12	5'-CCCCGGGC	35905
13	5'-GGGCGCGG	35858
14	5'-GCCCCGGG	35849
15	5'-GCCCGCGG	35751
16	5'-CGCCGCGC	35749
17	5'-GGGCGGGC	35581
18	5'-CCGCCCGG	35478
19	5'-GCCGGCGG	35130
20	5'-CCCGCCGG	34927

B

In bold is the sequence chosen for further study to compare to other nucleic acid sequences and structures.

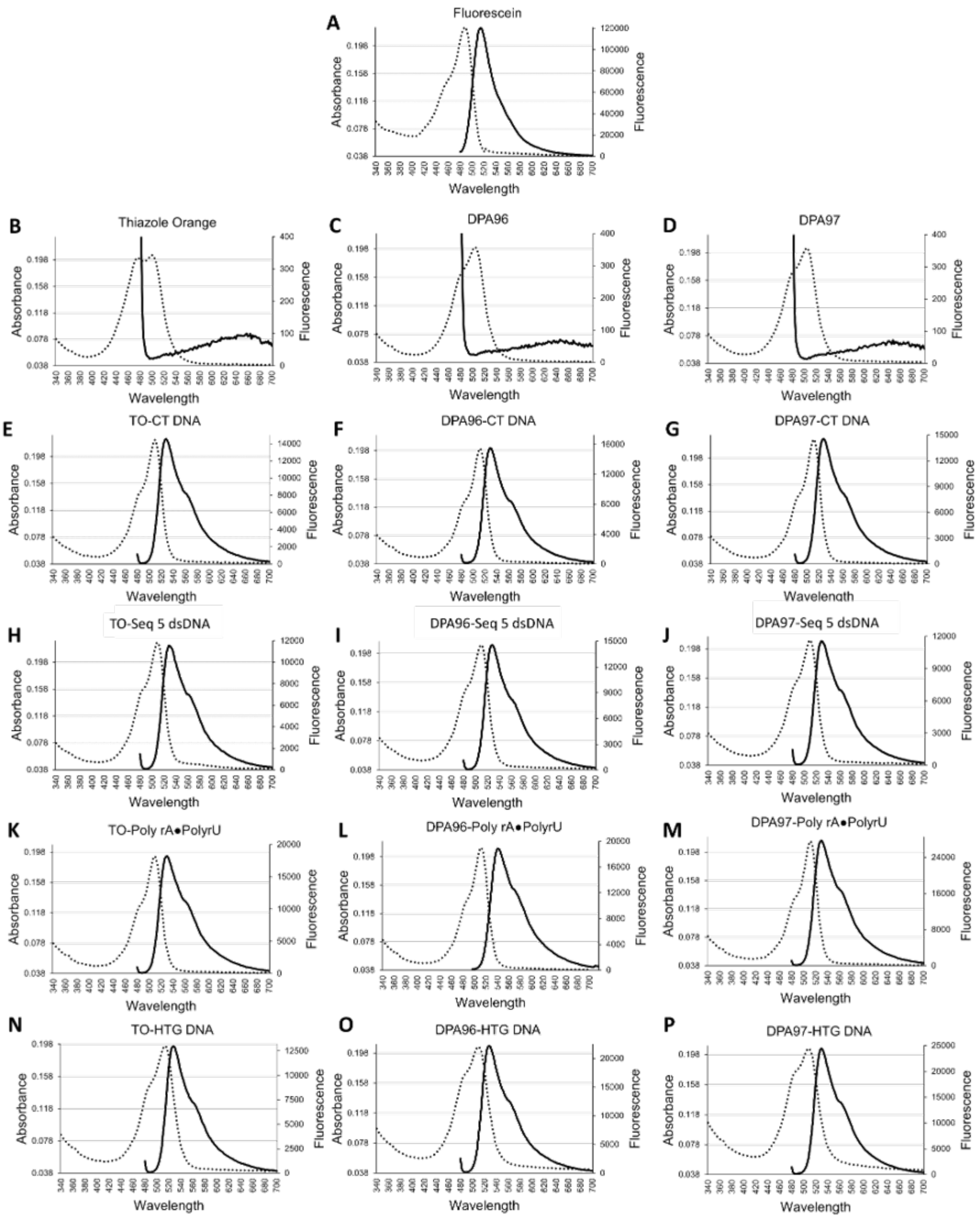

Figure S3. Absorbance and fluorescence emission spectra of (**A**) fluorescein, thiazole orange (**left column**), DPA96 (**middle column**), DPA97 (**right column**) into calf thymus DNA, Seq 5 dsDNA, and Poly (rA) ● Poly (rU). Assays were performed in HEPES buffer pH 7.0, 100 mM KCl, 0.4 mM EDTA. The concentration of calf thymus and duplexes was 10 μM. Fluorescence intensity values represent measurements recorded at 465 nm. Legend: The concentration range for nucleic acids was 1 – 16 μM. Fluorescence scans were performed in HEPES buffer pH 7.0, 100 mM KCl, 0.4 mM EDTA. The concentration of calf thymus and duplexes was 10 μM.

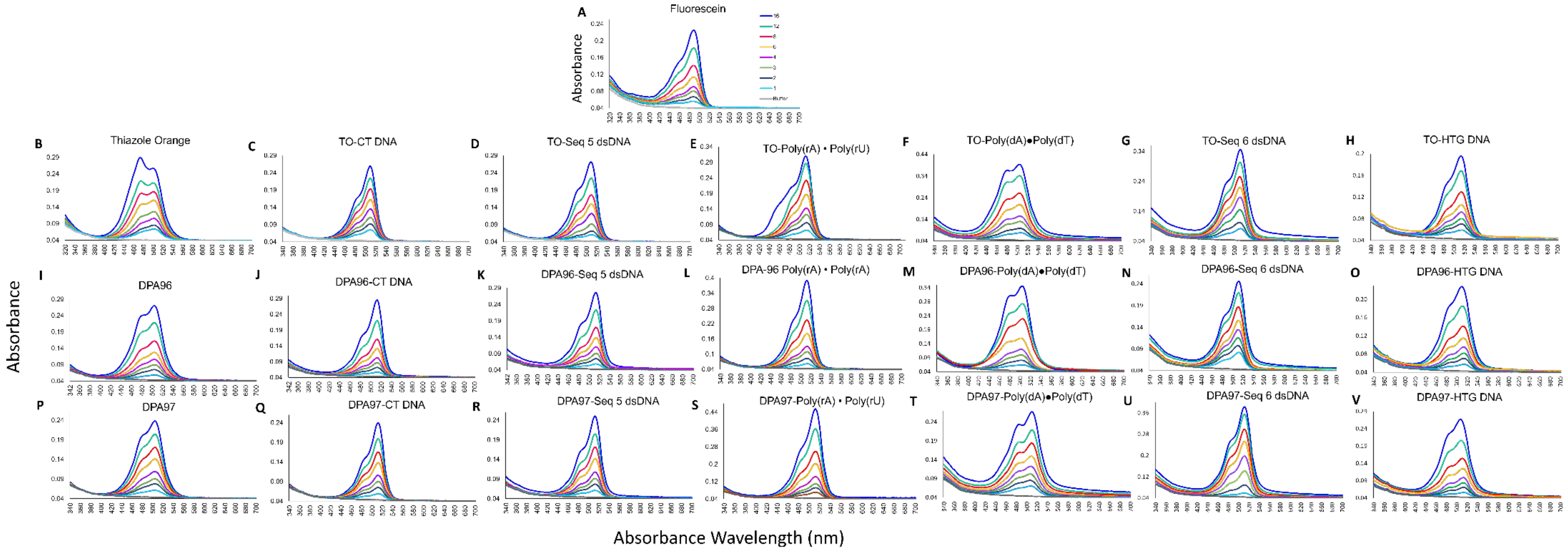

Figure S4. Absorbance scans for titration of (**A**) fluorescein, (**B**-**H**) thiazole orange, (**I**-**O**) DPA96, and (**P**-**V**) DPA97 into calf thymus DNA, Seq 5 dsDNA, and Poly(rA) ● Poly(rU), Poly(dA) ● Poly(dT), and Seq 6 dsDNA. Assays were performed in HEPES buffer pH 7.0, 100 mM KCl, 0.4 mM EDTA. The concentration for nucleic acids was 10 μM. Legend: The concentration range for TO and the TO derivatives was 1 – 16 μM.

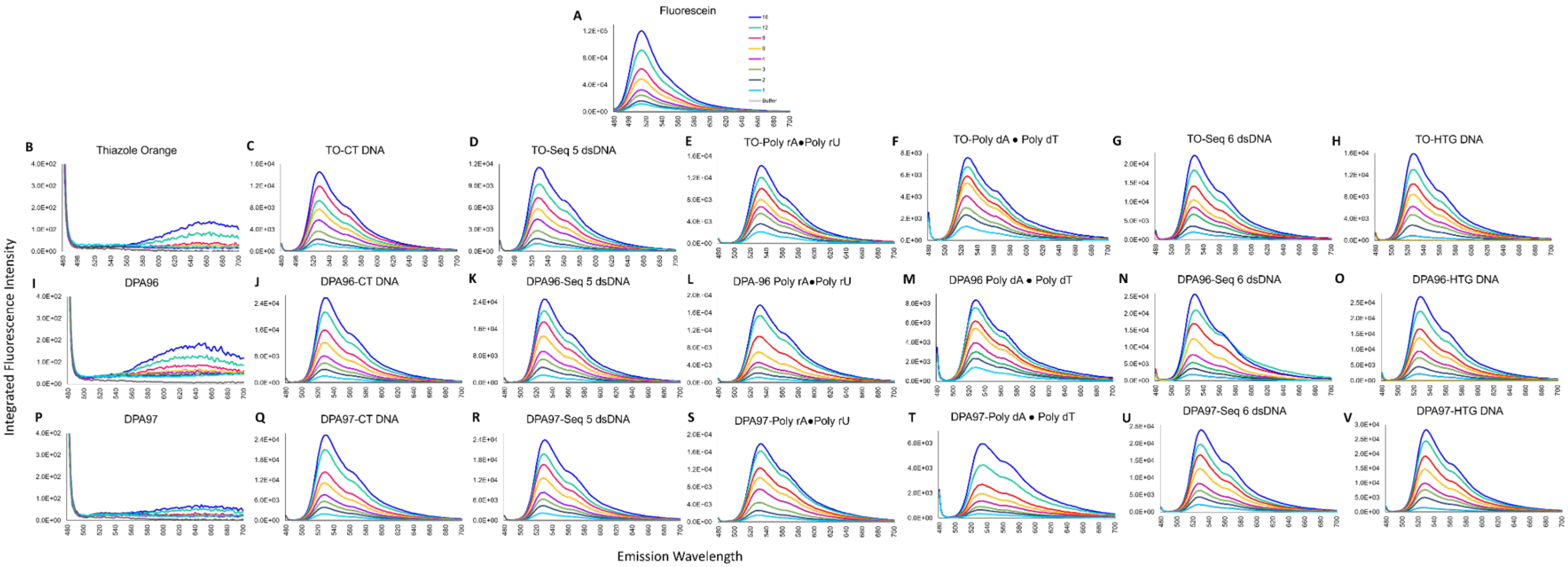

Figure S5. Fluorescence scans for titration of (**A**) fluorescein, (**B**-**H**) thiazole orange, (**I**-**O**) DPA96, and (**P**-**V**) into various nucleic acids. Assays were performed in HEPES buffer pH 7.0, 100 mM KCl, 0.4 mM EDTA. The excitation wavelength was 465 nm. Fluorescence scans were performed in HEPES buffer pH 7.0, 100 mM KCl, 0.4 mM EDTA. The concentration for nucleic acids was 10 μM. Legend: The concentration range for TO and the TO derivatives was 1 – 16 μM.

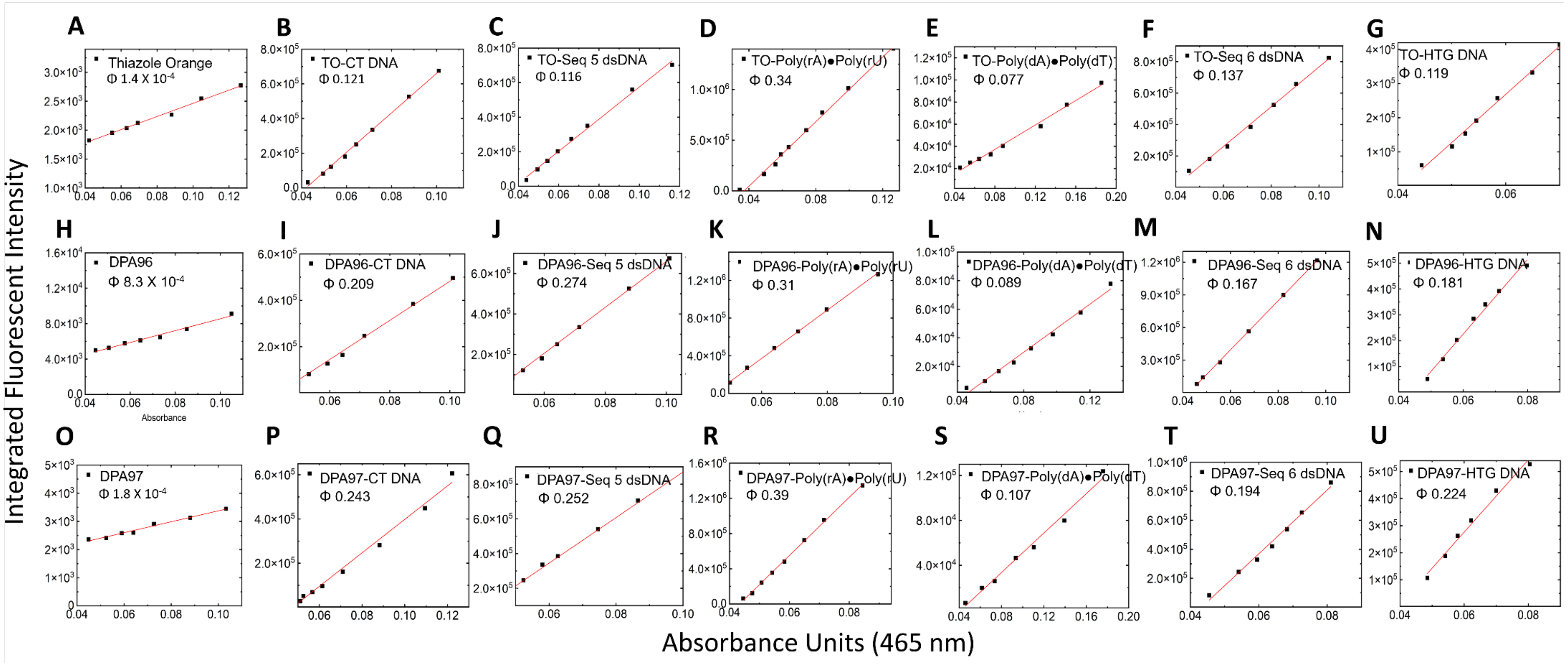

Figure S6. Linear fitted data plots of the integrated fluorescence intensity as a function of absorbance over the titration range of (**A**-**G**) thiazole orange, (**H**-**N**) DPA 96, (**O**-**U**) DPA 97 into various nucleic acids. Assays were performed in HEPES buffer pH 7.0, 100 mM KCl, 0.4 mM EDTA. The concentration of nucleic acids was 10 μM. Absorbance values represent measurements recorded at 465 nm.

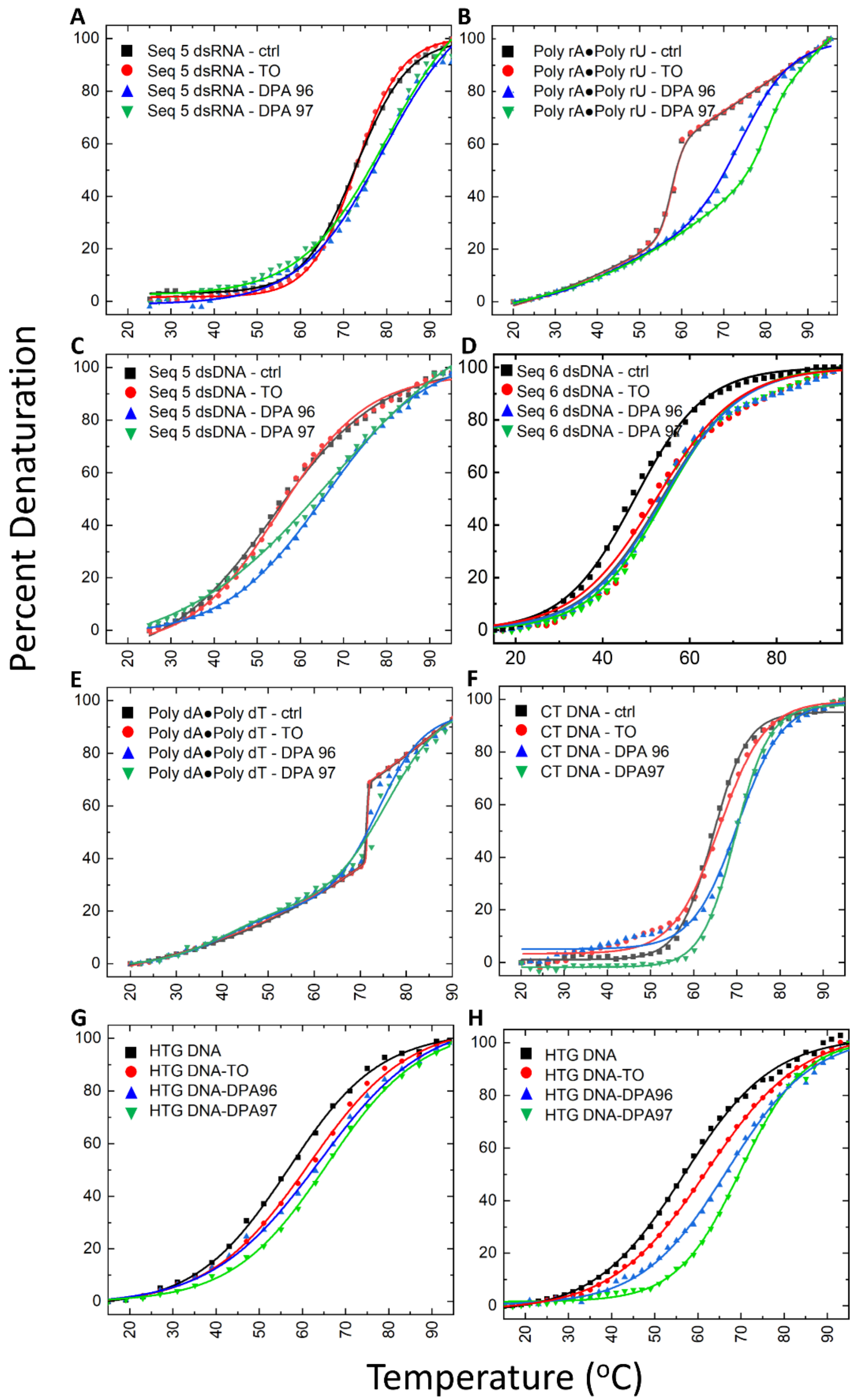

Figure S7. Thermal melting curves (**A**) Seq 5 dsRNA, (**B**) Poly rA ● Poly rU, (**C**) Seq 5 dsDNA, (**D**) Seq 6 dsDNA, (**E**) Poly dA ● Poly dT, (**F**) CT DNA, (**G**) HTG (1:10) ligand to DNA) and (**H**) HTG (1:1 ligand to DNA), without or in the presence of TO, DPA 96, or DPA 97. Thermal melting curves were performed in 10 mM HEPES buffer with 0.4 mM EDTA and 100 mM KCl for all except for CT DNA. CT DNA melting curves were done in 10 mM cacodylate with no additional salts. The concentration of the nucleic acid duplexes was 5 µM. Ligands were at a 1:10 ratio with nucleic acid (per base pair). A 1:1 ratio of ligand to HGT DNA was also tested.

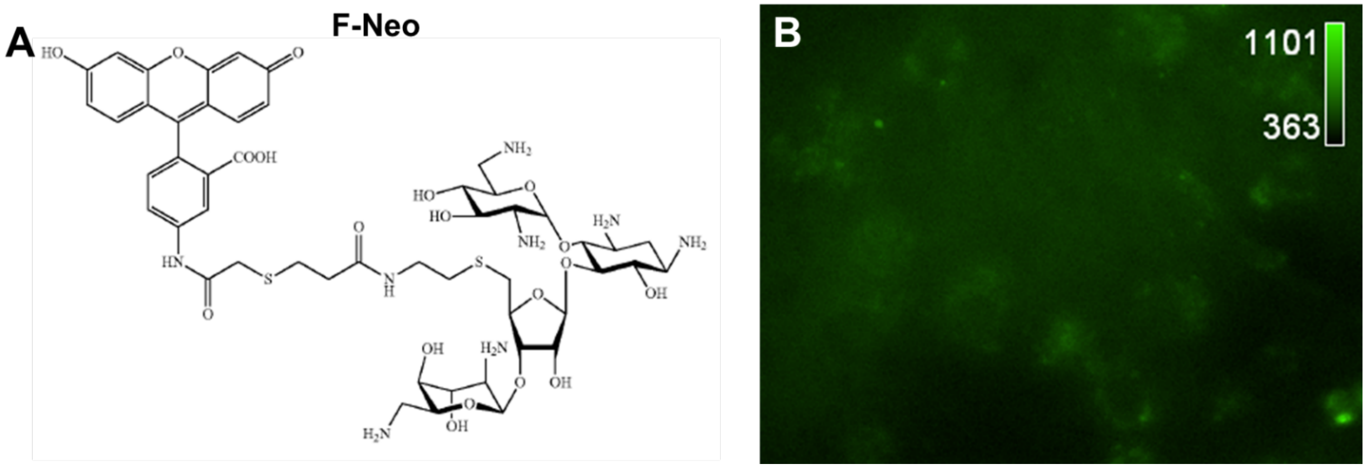

Figure S8. (**A**) Structure of F-Neo. (**B**) Epifluorescence microscopy image of J774A.1 macrophage cells after 24 hours of treatment with F-Neo.

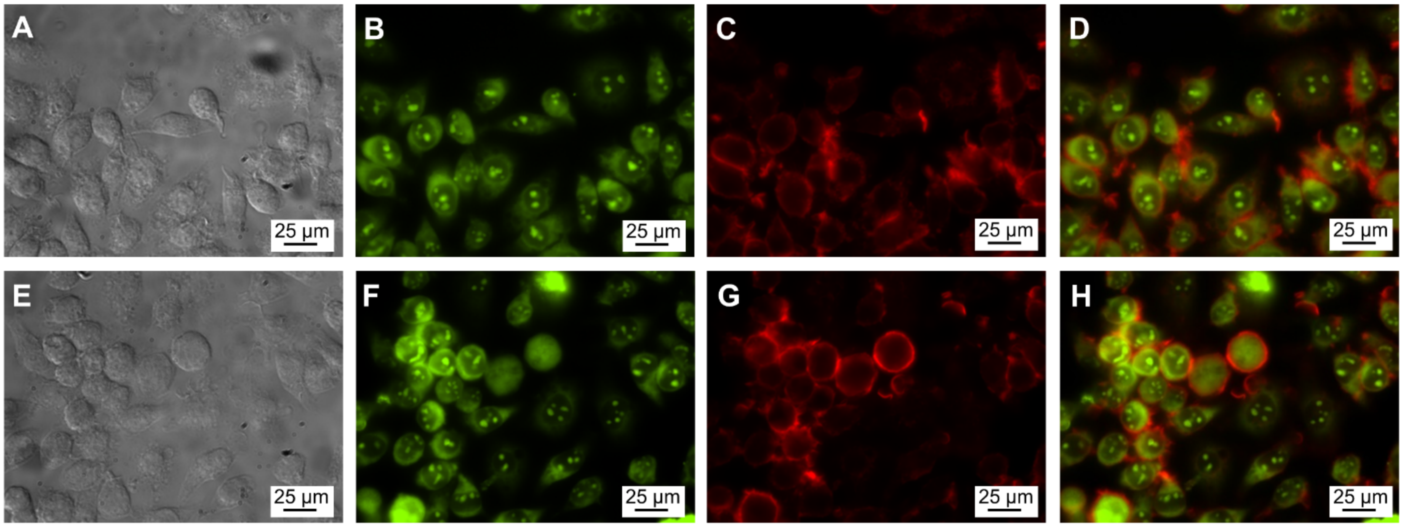

Figure S9. J774.A1 macrophage cells are treated with DPA 96 (**A**, **B**, **C**, **D**) or DPA 97 (**E**, **F**, **G**, **H**) and Alexa Fluor 594 phalloidin. DIC (**A**, **E**), FITC fluorescence filter (**B**, **F**), TRITC fluorescence filter (**C**, **G**), and DIC overlaid with the TRITC and FITC fluorescence filter overlaid (**D**, **H**). Images were taken at 60x magnification using a Nikon Ti-Eclipse microscope. Macrophage were treated with 5 µM TO-Neo for 20 minutes at 37°C in 5% CO_2.

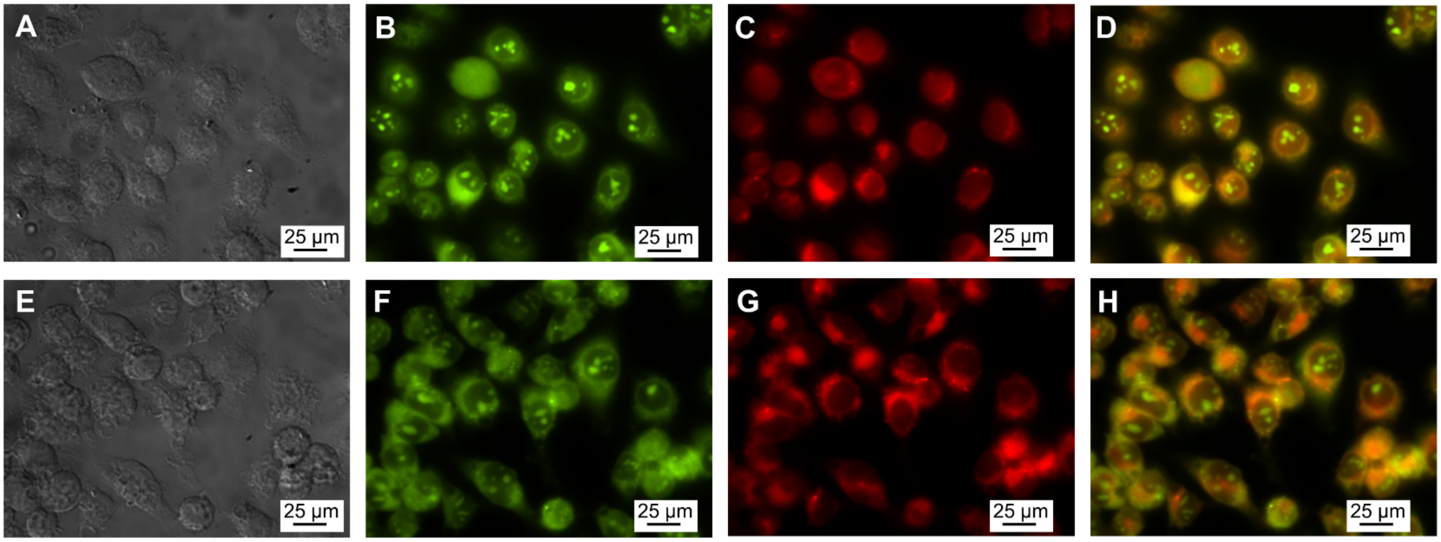

Figure S10. J774.A1 macrophage cells treated with DPA 96 (**A**, **B**, **C**, **D**) or DPA 97 (**E**, **F**, **G**, **H**) and MitoTracker. DIC (**A**, **E**), FITC fluorescence filter (**B**, **F**), TRITC fluorescence filter (**C**, **G**), DIC overlaid with the TRITC and FITC fluorescence filter overlaid (**D**, **H**). Images were taken at 60x magnification using a Nikon Ti-Eclipse microscope. Macrophage were treated with 5 µM TO-Neo for 20 minutes at 37°C in 5% CO_2.

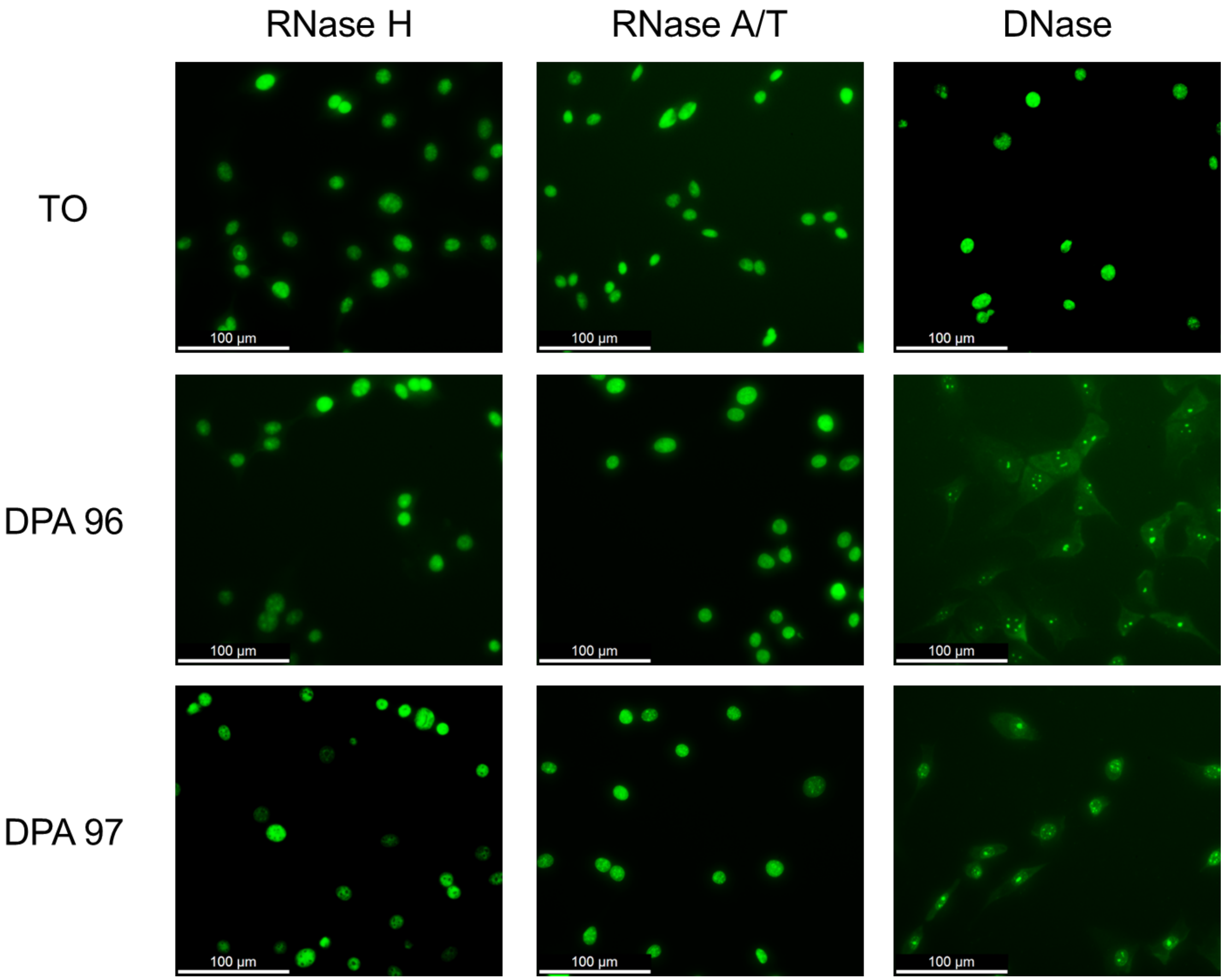

Figure S11. NIH-3T3 cells were fixed in pre-chilled methanol at -20°C for 1 min. The cell membrane was then permeabilized with 1% Triton X-100 in D-PBS for 2 min at room temperature. After rinsing with DPBS twice, the cells were incubated with 1.0 μM TO, DPA 96, or DPA 97 in D-PBS solution for 20 min at 37°C in a 5% CO_2 atmosphere, followed by washing with D-PBS buffer twice. DNase (100 U/mL), or RNase A/T1 (100 U/mL) was added into each of the three cells, which was then incubated at 37˚C in a 5% CO_2 atmosphere for 3 h. Cells were rinsed again by D-PBS buffer twice before imaging. The fluorescent imaging pictures were obtained in D-PBS buffer by using an equal exposure time for control, DNase, and RNase experiments.

Synthesis and Characterization of DPA 96

Scheme 1. Synthesis of DPA 96[a]

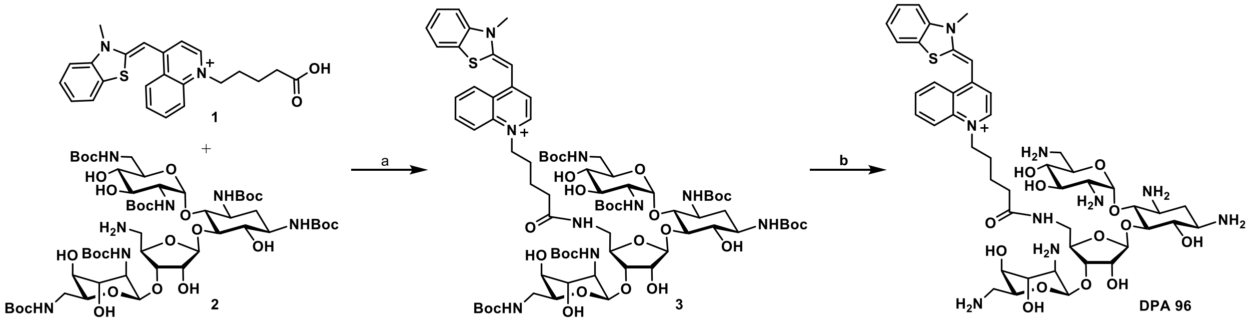

[a]Reagents and conditions: (a) HATU, DIPEA, DMF, 24 h, rt. (b) TFA/dichloromethane (1:1), 30 min, rt.

1-[4-({[(2*S*,3*R*,4*S*,5*R*)-5-[(1*S*,2*R*,3*S*,5*R*,6*S*)-3,5-Bis[*tert*-butyl(oxycarbonylamino)]-6-[(2*S*,3*S*,4*S*,5*R*,6*S*)-3-[*tert*-butyl(oxycarbonylamino)]-6-{[*tert*-butyl(oxycarbonylamino)]methyl}-4,5-dihydroxytetrahydro-2*H*-pyran-2-yloxy]-2-hydroxycyclohexyloxy]-3-[(2*S*,3*S*,4*S*,5*R*,6*R*)-3-[*tert*-butyl(oxycarbonylamino)]-6-{[*tert*-butyl(oxycarbonylamino)]methyl}-4,5-dihydroxytetrahydro-2*H*-pyran-2-yloxy]-4-hydroxytetrahydro-2-furyl]methyl}carbamoyl)butyl]-4-[(*Z*)-(3-methyl-2,3-dihydro-1,3-benzothiazol-2-ylidene)methyl]-1-quinolinium (3). To a solution of hexa-*N*-Boc-deoxy-neomycin-5″-amine **2** (30 mg, 25 μmol) in anhydrous DMF (3.0 mL), HATU (38 mg, 0.10 mmol, 4.0 equiv.) was added, followed by the addition of DIPEA (25 mg, 0.20 mmol, 8.0 equiv.) and aromatic carboxylic acid **1** (14 mg, 36 μmol, 1.4 equiv.). The reaction was stirred at room temperature for 24 h in the dark and monitored by TLC. The crude was directly purified by semi-prep HPLC (Agilent Infinity 1260, ZORBAX 300SB-*C*18, 9.4×250 mm, 4 mL/min) utilizing a 25 min gradient program of 0 to 100% ACN with 0.1% TFA in H_2O with 0.1% TFA. Fractions for the desired product were collected at 19.5 min and lyophilized to afford the desired product **3** as a deep red solid (4.0 mg, 10.2%). [R_f = 0.42, 10% MeOH in CH_2Cl_2 (v/v)]; LRMS (ESI/SQD) m/z: $[M+H]^+$ calcd. for $C_{76}H_{117}N_9O_{25}S$ = 1587.78; found 1587.71; HRMS (ESI/TOF) m/z: $[M]^+$ calcd. for $C_{76}H_{116}N_9O_{25}S$ = 1586.7803; found 1586.7831.

1-[4-({[(2*S*,3*R*,4*S*,5*R*)-3-[(2*S*,3*S*,4*S*,5*R*,6*R*)-3-Amino-6-(aminomethyl)-4,5-dihydroxytetrahydro-2*H*-pyran-2-yloxy]-5-[(1*S*,2*R*,3*S*,5*R*,6*S*)-3,5-diamino-6-[(2*S*,3*S*,4*S*,5*R*,6*S*)-3-amino-6-(aminomethyl)-4,5-dihydroxytetrahydro-2*H*-pyran-2-yloxy]-2-hydroxycyclohexyloxy]-4-hydroxytetrahydro-2-furyl]methyl}carbamoyl)butyl]-4-[(*Z*)-(3-methyl-2,3-dihydro-1,3-benzothiazol-2-ylidene)methyl]-1-quinolinium

(DPA 96). To a solution of **3** (4.0 mg, 2.5 μmol) in dichloromethane (1.0 mL), TFA (1.0 mL) was added. The solution was stirred at room temperature for 30 min in the dark. Then, dichloromethane was removed under

vacuum and diethyl ether was added to precipitate the product. The precipitated product was pelleted, decanted, and washed twice with diethyl ether to afford the desired product **DPA 96** (2.0 mg, 65%). ^{1}H NMR (500 MHz, D_2O) δ 8.18 (d, *J* = 8.4 Hz, 1H), 7.84 (dd, *J* = 7.4 Hz, 2.0 Hz, 1H), 7.67 (t, *J* = 7.9 Hz, 1H), 7.56-7.45 (m, 3H), 7.19 (t, *J* = 7.8 Hz, 1H), 7.07 (t, *J* = 7.3 Hz, 1H), 6.91 (d, *J* = 7.7 Hz, 2H), 5.78 (dd, *J* = 4.2 Hz, 2.0 Hz, 1H), 5.22 (dd, *J* = 4.0 Hz, 2.0 Hz, 1H), 5.08 (s, 1H), 4.17 (td, *J* = 5.2 Hz, 2.0 Hz, 1H), 4.07 (ddd, *J* = 16.9 Hz, 6.2 Hz, 2.7 Hz, 7H), 3.93 (t, *J* = 7.95 Hz, 1H), 3.87-3.72 (m, 3H), 3.64 (dt, *J* = 3.2 Hz, 1.6 Hz, 1H), 3.61-3.52 (m, 1H), 3.48-3.08 (m, 14H), 2.33 (dd, *J* = 11.3 Hz, 5.8 Hz, 1H), 2.19-2.12 (m, 2H), 1.71-1.64 (m, 2H), 1.48 (q, *J* = 7.9 Hz, 2H); LRMS (ESI/SQD) m/z: $[M+H]^+$ calcd. for $C_{46}H_{69}N_9O_{13}S$ = 987.47; found 988.41; $[M+2H]^+$ calcd. for $C_{46}H_{70}N_9O_{13}S$ = 494.23; found 494.69; HRMS (ESI/TOF) m/z: $[M]^+$ calcd. for $C_{46}H_{68}N_9O_{13}S$ = 986.4657; found 986.4669.

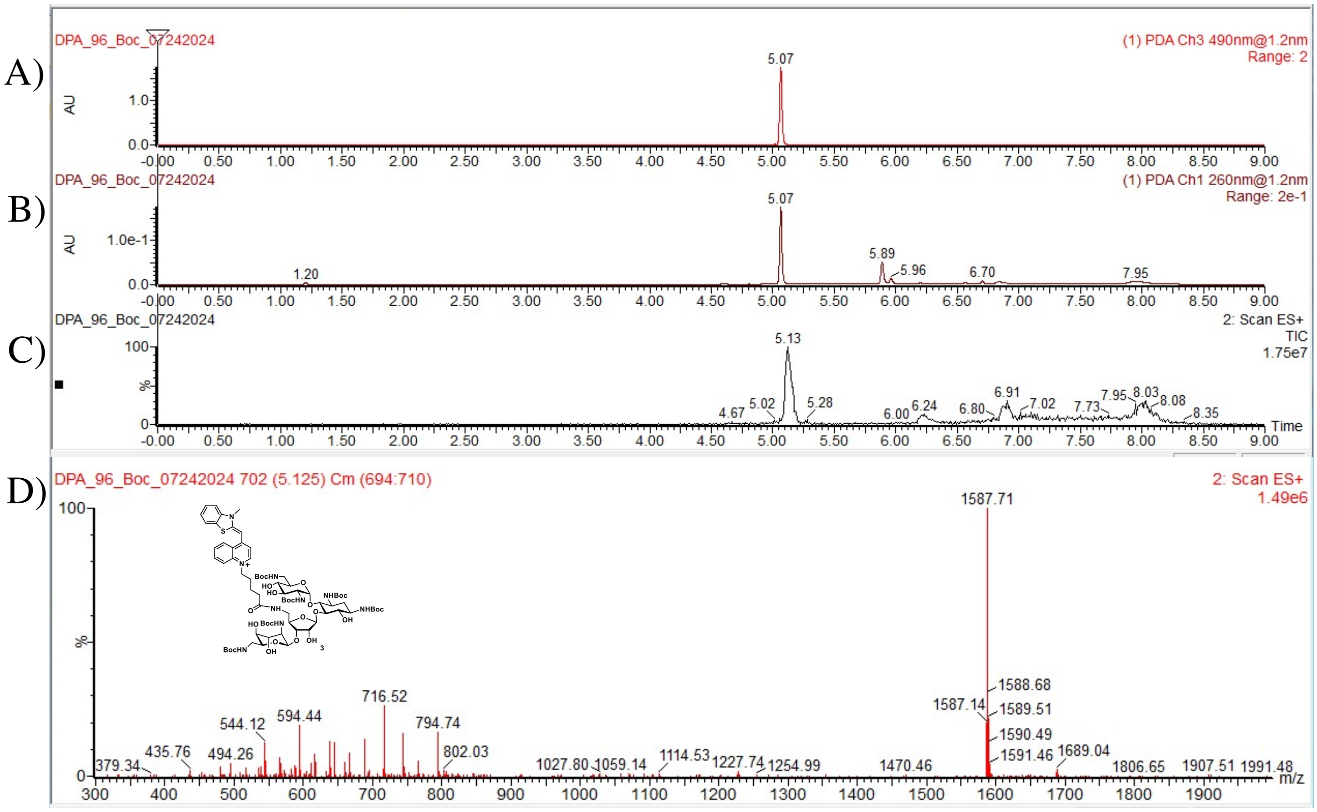

Figure S12. UPLC-MS (waters ACQUITY UPLC H-class PLUS, SQ detector 2) analysis of **3**. (A) UPLC profile at 490 nm. Purity is 100%. (B) UPLC profile at 260 nm. (C) Total ion chromatogram. (D) MS spectrum at 5.13 min. Column: Peptide CSH-*C*18 (2.1 x 100 mm, 1.7 µm). Flow rate: 0.3 mL/min. Solvent system: H_2O with 0.1% FA (A) and ACN with 0.1% FA (B). Gradient: 0-95% B in A over 8 min. LRMS (ESI/SQD) m/z: $[M+H]^+$ calcd. for $C_{76}H_{117}N_9O_{25}S$ = 1587.78; found 1587.71.

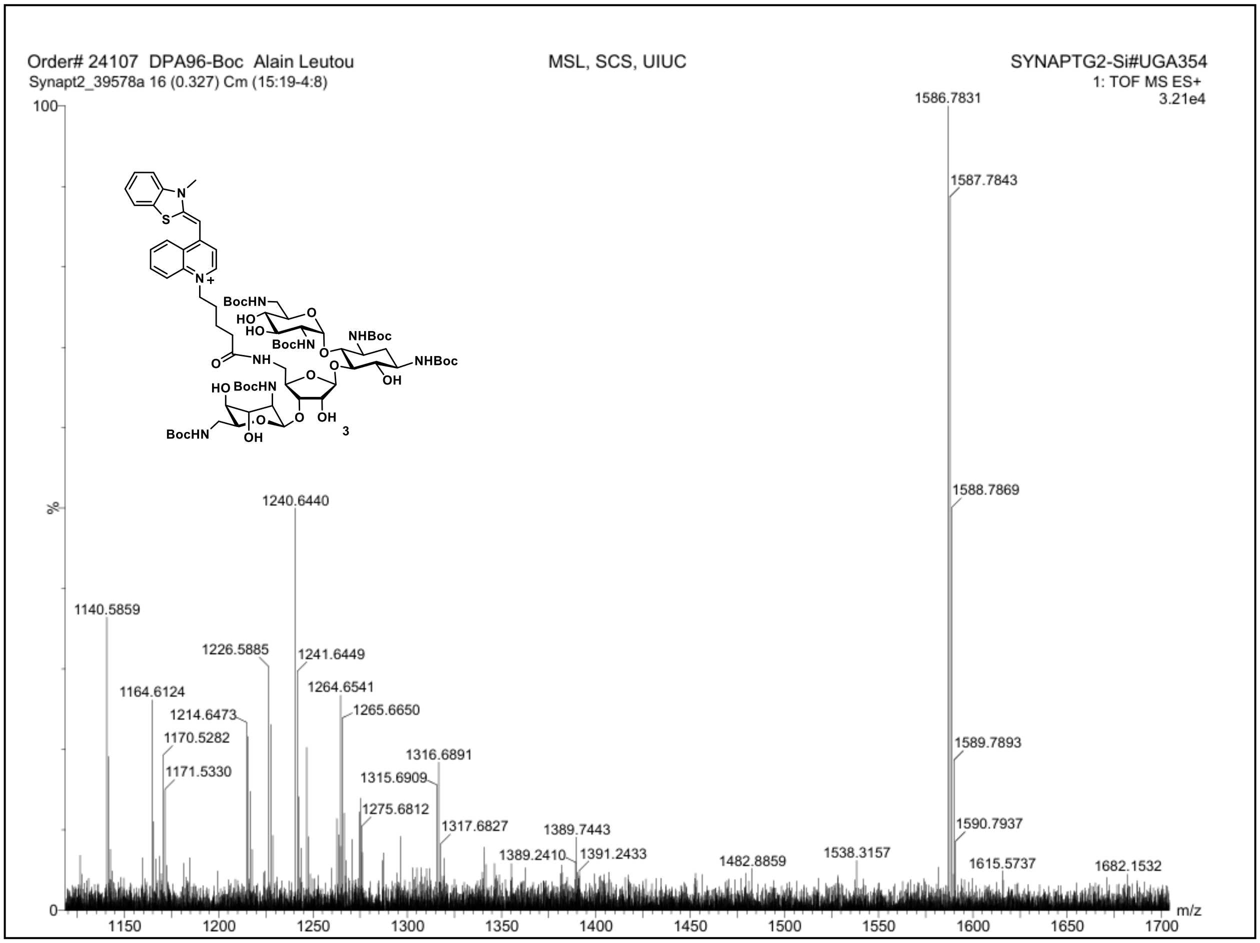

Figure S13. HRMS analysis of **3**. HRMS (ESI/TOF) m/z: $[M]^+$ calcd. for $C_{76}H_{116}N_9O_{25}S$ = 1586.7803; found 1586.7831.

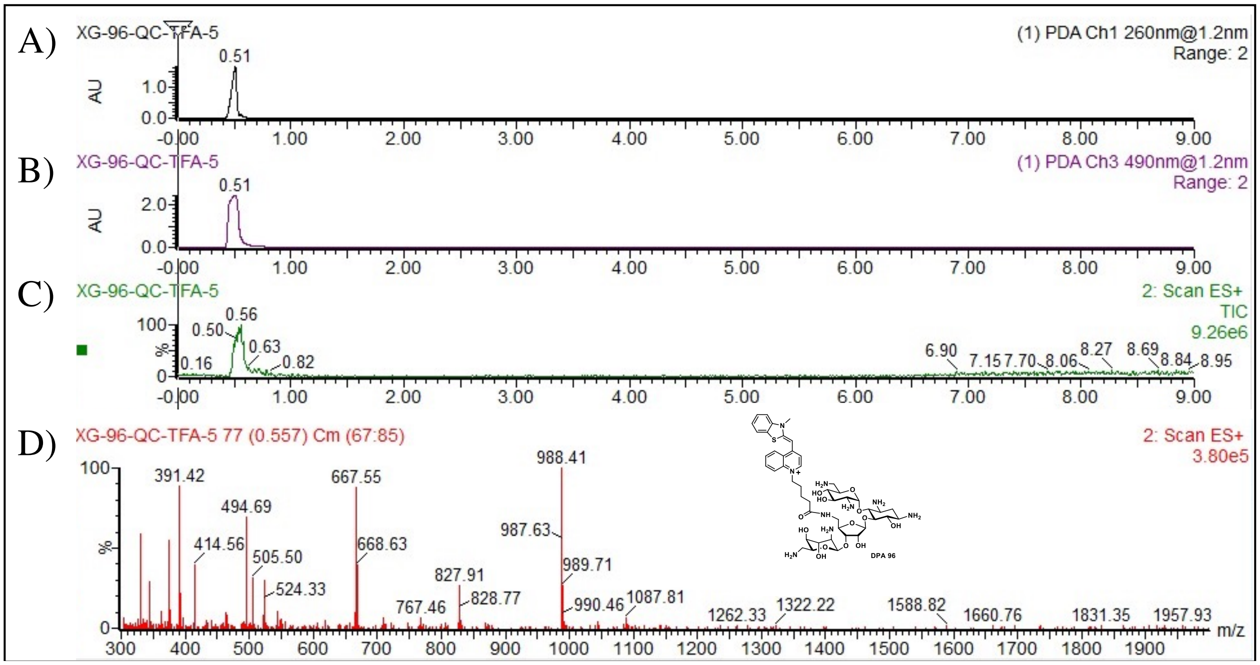

Figure S14. UPLC-MS (waters ACQUITY UPLC H-class PLUS, SQ detector 2) analysis of **DPA 96**. (A) UPLC profile at 260 nm. (B) UPLC profile at 490 nm. (C) Total ion chromatogram. (D) MS spectrum at 0.56 min. Column: Peptide CSH-*C*18 (2.1 x 100 mm, 1.7 µm). Flow rate: 0.3 mL/min. Solvent system: H_2O with 0.1% FA (A) and ACN with 0.1% FA (B). Gradient: 0-95% B in A over 8 min. LRMS (ESI/SQD) m/z: $[M+H]^+$ calcd. for $C_{46}H_{69}N_9O_{13}S$ = 987.47; found 988.41; $[M+2H]^+$ calcd. for $C_{46}H_{70}N_9O_{13}S$ = 494.23; found 494.69.

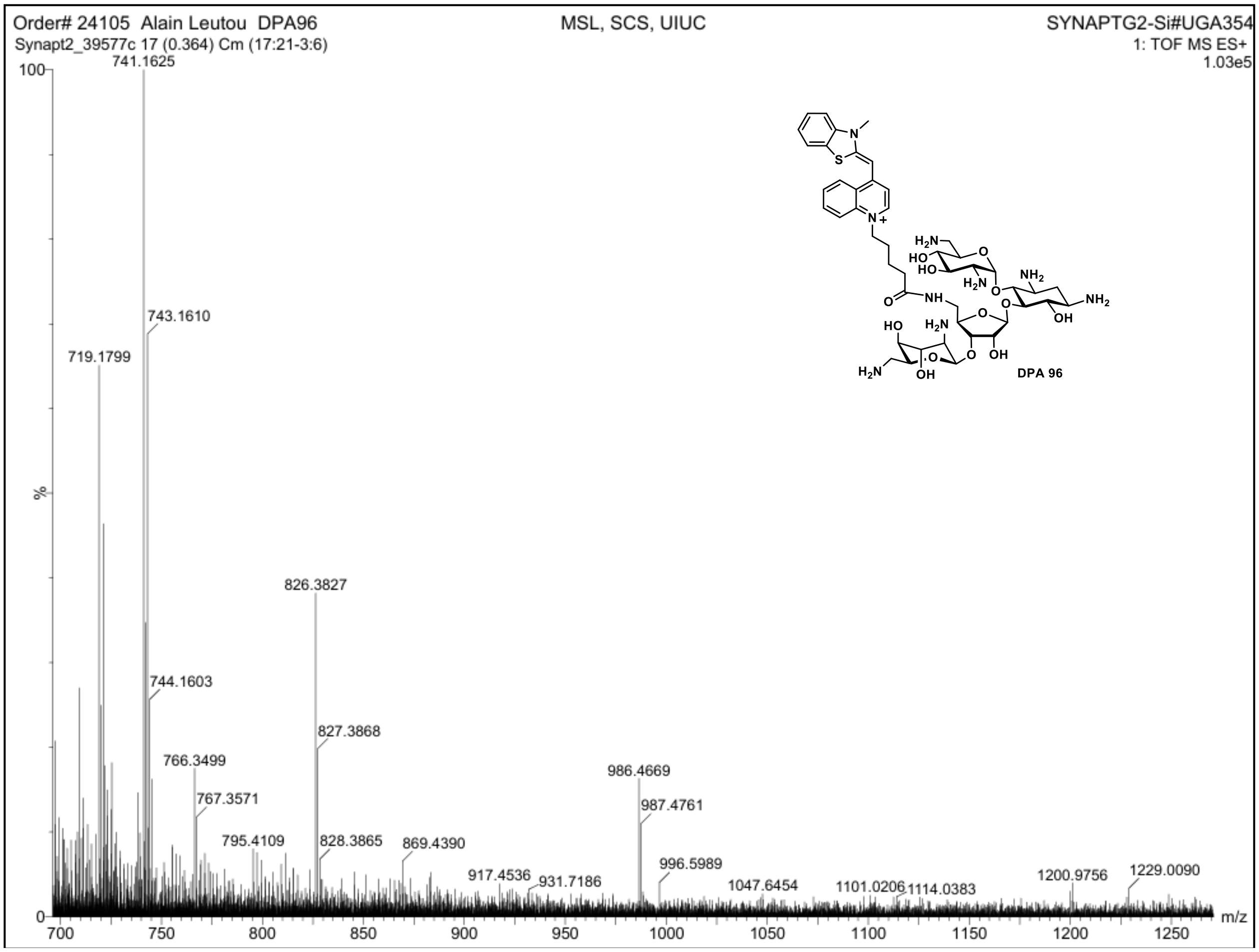

Figure S15. HRMS analysis of **DPA 96**. HRMS (ESI/TOF) m/z: $[M]^+$ calcd. for $C_{46}H_{68}N_9O_{13}S$ = 986.4657; found 986.4669.

A)

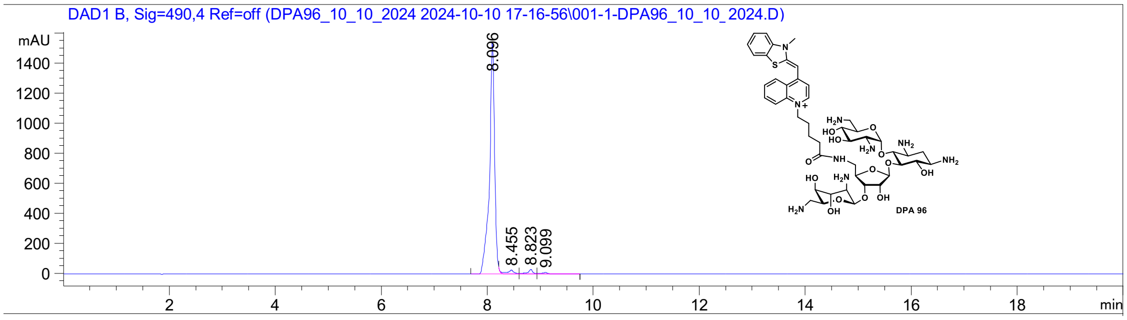

Signal 1: DAD1 B, Sig=490,4 Ref=off

Peak #	RetTime [min]	Type	Width [min]	Area [mAU*s]	Height [mAU]	Area %
→ 1	8.096	BV R	0.0999	1.05284e4	1537.73535	95.2534
2	8.455	VV E	0.1230	238.03818	25.96634	2.1536
3	8.823	VV E	0.0936	201.68686	30.32435	1.8247
4	9.099	VB E	0.1117	84.91413	10.35479	0.7682

B)

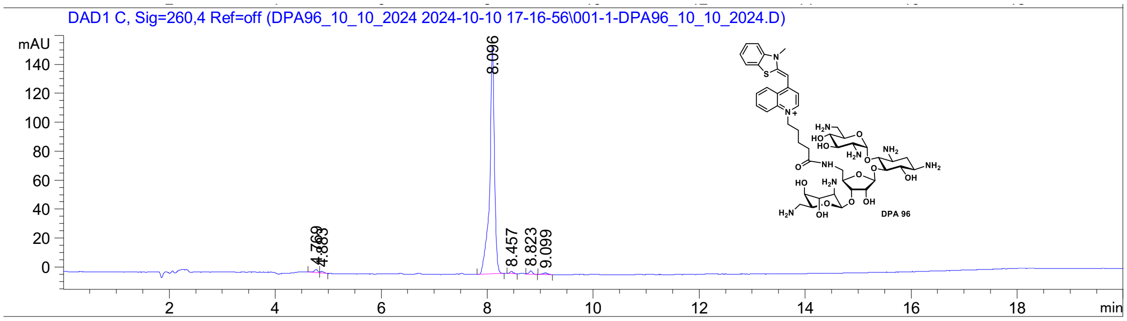

Signal 2: DAD1 C, Sig=260,4 Ref=off

Peak #	RetTime [min]	Type	Width [min]	Area [mAU*s]	Height [mAU]	Area %
1	4.769	BV	0.0718	10.77822	2.25538	1.0556
2	4.883	VB	0.0763	5.77108	1.15506	0.5652
→ 3	8.096	BB	0.0888	979.50629	156.97672	95.9346
4	8.457	BB	0.0644	6.65531	1.54159	0.6518
5	8.823	VB	0.0720	11.23198	2.33990	1.1001
6	9.099	BB	0.0935	7.07169	1.09307	0.6926

Figure S16. HPLC (agilent 1100) analysis of **DPA 96**. (A) HPLC profile at 490 nm. Purity is 95.3%. (B) HPLC profile at 260 nm. Purity is 95.9%. Column: Zorbax 300SB-*C*18 (4.6 x 150 mm, 5.0 µm). Flow rate: 1 mL/min. Solvent system: H_2O with 0.1% TFA (A) and ACN with 0.1% TFA (B). Gradient: 0-95% B in A over 20 min. HPLC purities were determined by integration of all signals.

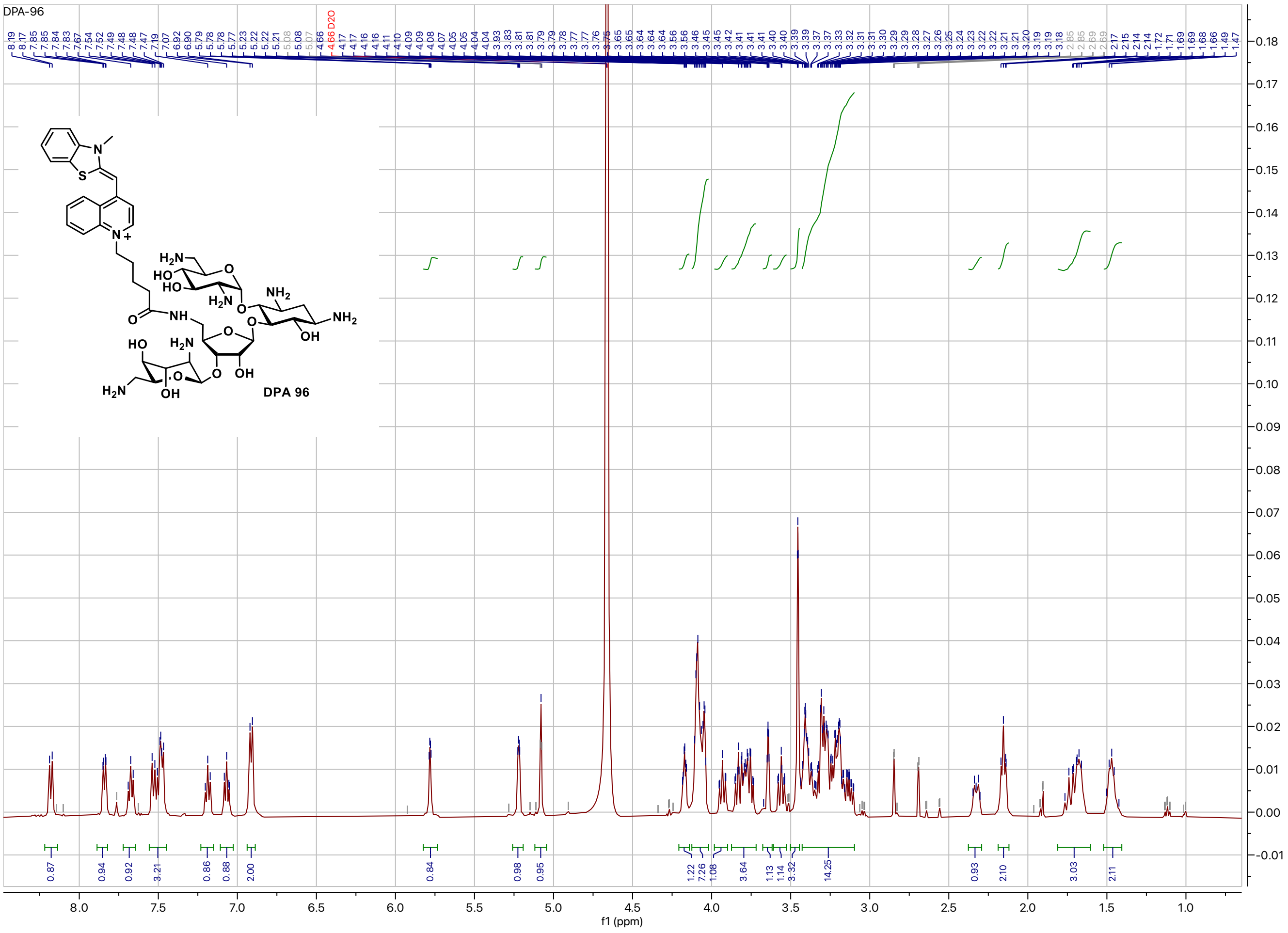

Figure S17. ^{1}H NMR of **DPA 96** (500 MHz, D_2O).
